# Activated Carbon Utilization from Corn Derivatives for High-Energy-Density Flexible Supercapacitors

**DOI:** 10.1021/acs.energyfuels.3c01925

**Published:** 2023-11-22

**Authors:** Kiran Kumar Reddy Reddygunta, Rachael Beresford, Lidija Šiller, Leonard Berlouis, Aruna Ivaturi

**Affiliations:** † Smart Materials Research and Device Technology (SMaRDT) Group, Department of Pure and Applied Chemistry, University of Strathclyde, Thomas Graham Building, Glasgow G1 1XL, U.K.; ‡ School of Engineering, Newcastle University, Newcastle upon Tyne NE1 7RU, U.K.

## Abstract

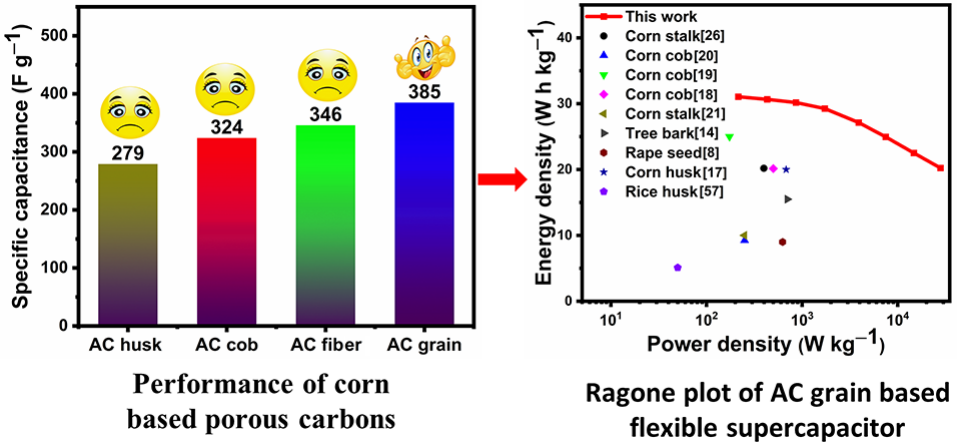

Porous activated carbons from four types of corn derivatives (husk, fiber, grain, and cob) are compared for the first time regarding their structural, morphological, and electrochemical characteristics for application as electrode materials in flexible supercapacitors. Benefiting from its hierarchical porous structure, appropriate amount of N and O functional groups, large specific surface area (1804 m^2^ g^–1^), and high degree of graphitization, the activated carbon from corn grains displayed the best electrochemical performance as an electrode material for supercapacitor applications; when tested in a three-electrode configuration, it had a high specific capacitance (411 F g^–1^ at 1.0 A g^–1^) and an excellent rate capacity (85.7% capacitance retention at 30 A g^–1^) in an aqueous 6 M KOH electrolyte. The high specific surface area and high degree of graphitization of the activated carbon from corn grains (AC grain) played crucial roles in its excellent energy storage performance. Most importantly, the flexible supercapacitor that was assembled with slot-die coated AC grain electrodes and a hydroxyethyl cellulose (HEC)/KOH biopolymer electrolyte delivered an outstanding electrochemical performance with an energy density of 31.1 Wh kg^–1^ at 215 W kg^–1^ and ultrahigh cyclic stability (91.3% capacitance retention after 10 000 cycles at a current density of 5 A g^–1^). Also, the assembled flexible supercapacitor maintained an energy density of 20.03 Wh kg^–1^ even under a high power density of 28.01 kW kg^–1^. These findings conclude that the porous carbon material obtained from corn grains has enormous potential as a high-performance electrode material for supercapacitors.

## 1 Introduction

In the last two decades, the demand for flexible and portable electronic devices has increased rapidly, which in turn has necessitated the development of efficient and cost-effective energy storage systems (ESS).^1^ Supercapacitors are considered novel energy storage devices that have the potential to drive flexible and wearable electronics. Supercapacitors possess promising electrochemical features, such as rapid charging and discharging, excellent cyclic stability, and superior energy and power densities, which are all attractive features for an efficient electrochemical energy storage device.^2,3^ Supercapacitors are divided into two categories based on their charge storage principles. Pseudocapacitors, which are fabricated using conductive polymers and transition metal derivatives (oxides, sulfides, etc.), primarily rely on fast and reversible redox interactions between the electrode and electrolyte to store charge. In contrast, electrochemical double-layer capacitors mainly rely on carbonaceous materials (graphene, carbon nanotubes, activated carbon, etc.) for the electrostatic accumulation of charges at the electrode/electrolyte interface.^4,5^ Among the wide range of carbonaceous materials, biomass-derived activated carbon has a highly competitive edge toward supercapacitor electrodes because of their hierarchical porous structure, tunable specific surface area (SSA), good conductivity, excellent capacitive features with high cyclic stability, and easy preparation process. Additionally, biomass as an activated carbon precursor is a renewable, sustainable, and eco-friendly resource.^6,7^

So far, activated carbons from various biomass sources such as rapeseed meal,^8^ water spinach,^9^ cotton stalk,^10^ cherry flowers,^11^ mango seeds,^12^ spruce bark, watermelon rind,^13^ tree bark,^14^ and many more have been explored and investigated for supercapacitor electrodes. Corn derivatives are the choice of biomass precursors in this work, as they are inexpensive and a readily available agricultural resource. According to a recent report from the United States Department of Agriculture (USDA), the global corn production for 2022/23 is 1161.86 million metric tons, out of which the United Kingdom alone produces 25 000 t of corn. Corn is harvested and sold in the form of corn kernels or whole corn (with the cob). Commercially, corn kernels and seeds are utilized in cooking or as a source of corn starch. The corn cob serves as a support structure for the kernels, while both the corn kernel and cob are wrapped in a leaflike substance called the husk. Despite the impact of open burning on the environment and public health, it remains the most popular disposal method for corn residues (husk, cob, and fibers), including those left behind after corn processing, because it is easy to do and saves time and money for farmers.^15,16^ Corn residues, a type of common biomass waste, can be used to produce porous carbon materials, which can further be used for electrodes in supercapacitors, thereby solving the environmental issues arising from burning residues.

Several studies have been completed on the utilization of corn derivatives such as corn husks,^17^ corn cobs,^18−20^ corn stalks,^21^ corn grains,^22^ and corn stover^23^ for producing high-SSA activated carbons for supercapacitor electrodes, as shown in Table 1. For instance, Yu et al.^24^ pyrolyzed corn stalk cores to produce high-SSA activated carbon (2350 m^2^ g^–1^), which showed a specific capacitance of 140 F g^–1^ at 1 A g^–1^ in an aqueous electrolyte. Similarly, Shi et al.^25^ prepared corn straw-based activated carbon via a KOH activation process, which displayed a high SSA of 1067 m^2^ g^–1^ and a high specific capacitance of 239 F g^–1^ in a 6 M KOH electrolyte. Moon et al.^22^ synthesized activated carbon from corn grains, and the corresponding device displayed a specific capacitance of 257 F g^–1^ in a 6 M KOH electrolyte. Lei et al.^26^ synthesized two-dimensional sheet-like carbon nanosheets from corn stalk biomass, which possessed a high SSA of 1736 m^2^ g^–1^ and delivered an excellent energy density of 20.2 Wh kg^–1^ at a power density of 398 W kg^–1^ when tested as an electrode for a flexible supercapacitor device. Similarly, Zhang et al.^18^ converted corn cob to a high-SSA activated carbon material (1471 m^2^ g^–1^), which showed an excellent capacitance of 293 F g^–1^ at 1 A g^–1^ in a 6 M KOH electrolyte. Moreover, the assembled device with the same 6 M KOH electrolyte exhibited an energy density of 20.2 Wh kg^–1^ at a power density of 500 W kg^–1^ and retained 99.9% of its performance after 4000 cycles. It is clear from these publications that the researchers focused primarily on one specific corn residue as a source for the creation of activated carbon. Although many corn residues have been employed to make high-performance activated carbons for use in supercapacitors, no study has yet identified the ideal precursor for the synthesis of activated carbon.

**Table 1:** Electrochemical Performance of Corn-Derived Activated Carbons for Supercapacitor Applications Recently Reported in the Literature

		three-electrode measurements		device performance				
corn residue	SSA (m^2^ g^–1^)	electrolyte	specific capacitance (F g^–1^)	electrolyte	energy density (Wh kg^–1^)	power density (W kg^–1^)	cyclic stability	ref
corn cob	1288.0	1 M H_2_SO_4_	340.8 at 5 mV s^–1^	–	–	–	–	(32)
corn straw	1067.1	6 M KOH	239 at 1 A g^–1^	–	–	–	–	(25)
corn stalk core	2349.8	–	140 at 1 A g^–1^	–	–	–	–	(24)
corn starch	1239	6 M KOH	144 at 0.625 A g^–1^	–	–	–	–	(33)
corn cob	1471.4	6 M KOH	293 at 1 A g^–1^	6 M KOH	20.15	500	99.5%, 4000 cycles at 2 A g^–1^	(18)
corn cob	800	1 M H_2_SO_4_	390 at 0.5 A g^–1^	EMIMBF_4_	25	174	54%, 500 cycles at 0.1 A g^–1^	(19)
corn stalk	2152	6 M KOH	350.4 at 0.2 A g^–1^	1 M Na_2_SO_4_	10.01	249.9	99.8%, 10 000 cycles at 5 A g^–1^	(21)
corn cob	2508	6 M KOH	560 at 0.5 A g^–1^	6 M KOH	9.24	250	96.8%, 10 000 cycles at 1 A g^–1^	(20)
corn stalk	1736	–	–	1 M Na_2_SO_4_	20.2	398	92%, 10 000 cycles at 5 A g^–1^	(26)
corn husk	1370	–	–	1 M TEABF_4_/AN	20	248	90%, 5000 cycles at 2 A g^–1^	(17)
corn stover	10.9	6 M KOH	242 at 0.05 A g^–1^	6 M KOH	9.9	2500	92%, 2500 cycles at 1 A g^–1^	(23)

Additionally, it can be observed from the literature reports summarized in Table 1 that the supercapacitor devices that were made with activated carbon electrodes derived from corn residues were evaluated either in ionic liquid electrolytes (1-ethyl-3-methylimidazolium tetrafluoroborate (EMIMBF_4_) or 1 M tetraethylammonium tetrafluoroborate in acetonitrile (1 M TEABF_4_/AN)) or in aqueous electrolytes (6 M KOH, 1 M H_2_SO_4_, or 1 M Na_2_SO_4_). Although aqueous electrolytes offer a high electrochemical performance, their main drawbacks are their volatility and narrow electrochemical potential window, which puts restriction on the energy density of the device.^27^ For example, Wang et al.^20^ prepared a high-SSA porous carbon material (2508 m^2^ g^–1^) through KOH activation using corn cob as the precursor and reported the highest specific capacitance of 560 F g^–1^ at a current density of 0.5 A g^–1^ in a 6 M KOH electrolyte. However, this high-SSA corn cob-based activated carbon exhibited a smaller energy density of only 9.24 Wh kg^–1^ in the 6 M KOH electrolyte due to the narrow potential window (1 V) provided by the aqueous electrolyte. Yu et al.^26^ fabricated flexible supercapacitor devices using a synthetic polymer-based PVA/KOH gel electrolyte and corn stalk-derived activated carbon electrodes, which showed an areal capacitance of 136 mF cm^–2^ at 0.5 mA cm^–2^; however, the potential window of this device was limited to only 1 V. Although synthetic polymers function admirably when employed as supercapacitor electrolytes, the majority of them are nondegradable. Ionic liquids, in contrast, have several potential advantages, including good conductivity and ion mobility, a wide electrochemical window, negligible volatility, and nonflammability.^28,29^ Karnan et al.^19^ prepared corn cob-based activated carbon with a high SSA of 800 m^2^ g^–1^ via a chemical activation method, and the maximum energy density of the device prepared with this corn cob-derived activated carbon reached up to 25 Wh kg^–1^ at a power density of 174 W kg^–1^ in an EMIMBF_4_ electrolyte. Similarly, a high energy density of 20 Wh kg^–1^ was reported for a supercapacitor with corn husk-based activated carbon electrodes in a 1 M TEABF_4_/AN organic electrolyte.^17^ Even though the supercapacitor devices with ionic liquid and organic electrolytes showed higher energy densities and large operating voltage windows, the majority of ionic liquid and organic electrolytes have a number of significant limitations (such as high viscosity, poor ionic conductivity, and high costs) that can restrict their usefulness for supercapacitor applications on a large scale.^30^ Hence, there is a need for low-cost, eco-friendly solid-state biopolymer electrolytes with a strong ionic conductivity, broad potential window, minimal volatility and flammability, and superior thermal and electrochemical stability. Biopolymer (e.g., cellulose, agarose, chitosan)-based hydrogels have been proposed as an appealing solution for flexible and intelligent electrochemical energy storage systems due to their affordability and biodegradability.^31^

Thus, in the present study, activated carbons derived from different corn derivatives were investigated for their potential applications as electroactive materials to fabricate solid-state flexible supercapacitors with a HEC/KOH (hydroxyethyl cellulose/potassium hydroxide) biopolymer gel electrolyte as the ionic transport medium. The structural, compositional, and morphological features of the activated carbons obtained from the various corn derivatives (i.e., corn cob, corn husk, corn fiber, and corn grains) were compared using gas sorption analysis, X-ray diffraction (XRD), Raman spectroscopy, X-ray photoemission spectroscopy (XPS), and field emission scanning electron microscopy (FESEM) to obtain the optimal precursor for fabricating flexible supercapacitors. According to the analysis results, we conclude that the activated carbon from corn grains possessed a hierarchical porous structure with the highest specific surface area, a higher degree of graphitization, and a higher carbon content with a small amount of O and N heteroatoms (∼6% of O and N in this work) compared to the other activated carbons. Benefiting from these physicochemical properties, the activated carbon from corn grains (AC grain) delivered a high specific capacitance of 411 F g^–1^ at 1 A g^–1^ current density and an outstanding rate capability in a 6 M KOH electrolyte. Because none of the previous reports in the literature employed biopolymer electrolytes, we used hydroxyethyl cellulose (HEC)/KOH as the gel electrolyte for fabricating a flexible supercapacitor. The symmetric supercapacitor fabricated with AC grains electrodes and the HEC/KOH electrolyte displayed outstanding energy and power densities of 31.1 Wh kg^–1^ and 215 W kg^–1^, respectively. Furthermore, the as-prepared flexible supercapacitor retained 91.3% of its initial performance after 10 000 cycles at a current density of 5 A g^–1^. To the best of our knowledge, this is the first effort to conduct a comprehensive analysis of the activated carbons synthesized from various corn parts for use in supercapacitors.

## 2 Experimental Section

### 2.1 Materials and Methods

#### 2.1.1 Materials and Reagents

Corn was purchased from a local supermarket in Glasgow, U.K. Potassium bicarbonate (KHCO_3_, 99.7%), sodium sulfate (Na_2_SO_4_), potassium hydroxide (KOH), polyvinylidene fluoride (PVDF) binder, 1-methylpyrrolidine, and hydroxyethyl cellulose (HEC) were purchased from Sigma-Aldrich. Hydrochloric acid (HCl, 37%) was purchased from Fisher Scientific. All of the chemicals were used as received without any further purification.

#### 2.1.2 Preparation of Activated Carbon from Different Parts of Corn

The initial step of this research was to synthesize activated carbon from different corn derivatives. For this purpose, corn husk, corn cob, corn grains, and corn fiber were separated and dried at 100 °C for 48 h. A FRITSCH ball mill was employed to ground these corn materials into powders, and the fine powders with a particle size of ≤100 μm were collected by sieving through 100 μm mesh. The obtained powders were stored inside a hot air oven at 100 °C until further use.

The corn-based activated carbon was synthesized via a carbonization and activation process. First, the pulverized powder from a corn derivative (i.e., husk, cob, grain, or fiber) was placed in a tube furnace, and the temperature was ramped to 400 °C at a heating rate of 10 °C min^–1^ under nitrogen flow. The powder was kept at this temperature for 2 h to allow the carbonization process to take place.^24^ Then, the furnace was turned off and the powder was allowed to cool naturally for 2 h. The carbonized powder from the corn derivative was then subjected to chemical activation, in which the powder was thoroughly mixed with KHCO_3_ in a weight ratio of 1:3 using a mortar and pestle. After being mixed, the mixture was heat-treated at 900 °C for 2 h under a nitrogen atmosphere. The furnace was turned off and the mixture was allowed to cool naturally after the activation phase. The activated sample was removed from the tube furnace and repeatedly washed with 1 M HCl and deionized (DI) water until the pH of the filtrate was neutral. The activated sample was then rinsed with ethanol and dried at 100 °C overnight. The dried powder was collected and then stored prior to further characterization. The samples from the different corn parts are termed as AC grain, AC husk, AC fiber, and AC cob.

### 2.2 Materials Characterization

X-ray diffraction patterns of the synthesized materials were recorded using a Bruker D2 PHASER system by employing monochromatic Cu Kα radiation (λ = 1.5406 Å). The substrates were set to a rotation speed of 8 degrees per minute throughout the measurements. The SSA and pore volume of the as-prepared samples were calculated using the Brunauer–Emmett–Teller (BET) method and nonlinear density functional theory (NLDFT) by employing a Micromeritics ASAP 2020 porosity analyzer at 77 K. The samples were degassed for 3 h in a dynamic vacuum at a temperature of 300 °C prior to the SSA investigations. Raman spectra were recorded on a WITec Raman microscope (using a laser power of 2.69 mW, a wavelength of 532 nm, an acquisition time of 10 s, and a 100× objective lens). A FEI Quanta 250 FEGSEM instrument with a 5 kV electron beam was employed to record the morphology of the as-prepared carbon samples. High-resolution transmission electron microscopy (HRTEM) measurements were recorded on an FEI Titan Themis instrument operating at 200 kV and equipped with a CEOS DCOR probe corrector, a SuperX energy dispersive X-ray spectrometer (EDX), and a 4k × 4k Ceta CMOS camera. A Thermo Scientific Kα X-ray photoelectron spectrometer (East Grinstead, U.K.) was used for the X-ray photoemission spectroscopy (XPS) studies. High-resolution photoemission spectra of specific elemental areas (C 1s, O 1s, and N 1s) were acquired with a hemispherical electron analyzer using a pass energy of 40 eV and an energy step size of 0.05 eV. The spectra were taken with a monochromatic Al Kα X-ray source with an output energy of 1486.6 eV and a maximum X-ray beam spot size of 400 μm. A low energy dual-beam electron/ion flood cannon was used to compensate for the surface charge. All of the XPS and Raman spectra were normalized, and Fityk software was used to deconvolve the spectra using Voigt fitting.

### 2.3 Electrode Preparation for the Three-Electrode and Two-Electrode Measurements

For the three-electrode measurements, a homogeneous slurry was prepared by mixing the electroactive material (i.e., the activated carbon (AC) from corn grain, corn husk, corn fiber, or corn cob) and PVDF in a weight ratio of 90:10, along with a few drops of 1-methylpyrrolidine. The slurry was then coated on 1 cm^2^ area stainless steel mesh (3 cm × 1 cm) using doctor blading and dried at 80 °C for 2 h. The stainless steel mesh had an aperture of 0.026 mm and a wire diameter of 0.025 mm. The weight of the activated carbon on the stainless steel mesh electrodes was as follows: AC grain = 2.4 mg, AC fiber = 2.5 mg, AC cob = 2.8 mg, and AC husk = 2.5 mg.

For the two-electrode measurements (i.e., for a flexible supercapacitor), electrodes were prepared using an Ossila slot-die coater (L2005A1). For this purpose, activated carbon ink was prepared according to the following procedure. First, 20 mg of AC grain was mixed with 15 wt % of Nafion and dispersed in isopropyl alcohol (IPA) under sonication for 4 h. The activated carbon ink was coated on top of the stainless steel mesh (l × w × t = 4.5 cm × 2 cm × 0.07 cm) using the slot-die coater. For the purpose of slot-die coating, 5 mL of the AC grain ink was loaded into the syringe and assembled with the slot-die head, faced the stainless steel mesh placed on the hot plate. The hot plate was preset to 80 °C before the coating was initialized. The AC grain ink was slot-die coated onto the stainless steel mesh current collector with a coating speed of 2.1 mm s^–1^ and a dispense rate of 1.1 μL s^–1^ over an area of 6 cm^2^ (l × w = 3 cm × 2 cm). After each coating, the mesh electrode was allowed to dry on the hot plate at 80 °C for 10 min before successive coatings were applied. The process was repeated 5 times so that a homogeneous film was formed on top of the stainless steel mesh. Finally, two slot-die-coated carbon electrodes with an area of 6 cm^2^ (l × w = 3 cm × 2 cm) were used to fabricate the solid-state supercapacitor.

The device was finally assembled by sandwiching the slot-die-coated carbon electrodes with a gel polymer electrolyte. The HEC/KOH gel electrolyte was prepared by dissolving 2 g of HEC in 20 mL of DI water, which was stirred continuously at 90 °C for 1 h. Then, 2 g of KOH (dissolved in 10 mL of DI water) was added dropwise to the HEC mixture over the course of 15 min, and the resulting mixture was stirred further for 1 h at 90 °C. A transparent HEC/KOH gel electrolyte was obtained after 2 h, which was placed inside a desiccator overnight to remove any air bubbles in the gel. The HEC/KOH gel electrolyte was then applied to both electrode surfaces and allowed to dry overnight within the fume hood at room temperature. Thus, on the surface of both electrodes, a thin, white HEC/KOH layer was formed. The electrodes with their gel coatings were then neatly sandwiched together without a separator and tightly sealed. To increase the contact between the electrode/electrolyte interface, the sealed device was pressed firmly using a pellet press machine (Atlas 15 ton manual hydraulic press) under an applied pressure of 1 ton for 5 min.

### 2.4 Electrochemical Measurements

The electrochemical behavior of AC grain, AC husk, AC fiber, and AC cob was studied by cyclic voltammetry (CV), galvanostatic charge–discharge (GCD) tests, and electrochemical impedance spectroscopy (EIS). All of the CV, GCD, and EIS experiments were run on an Autolab PGSTAT302N potentiostat/galvanostat with the FRA32M module at room temperature. The working, reference, and counter electrodes for a typical three-electrode measurement were activated carbon-coated stainless steel mesh, Ag/AgCl, and platinum wire, respectively. Either 1 M Na_2_SO_4_ or 6 M KOH was used as the electrolyte for the three-electrode measurements here.

The specific capacitance *C*_s_ (F g^–1^) was calculated from the GCD curves in the three-electrode configuration using the following equation:^34^
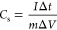
1where *I* is the current (A), Δ*t* is the discharge time after the *IR* drop, *m* is the weight of the active material on the mesh electrode, and Δ*V* is the operating voltage window after the *IR* drop.

The electrochemical performance of the flexible supercapacitor device with the HEC/KOH electrolyte was tested in a two-electrode configuration. The total capacitance *C*_t_ (F g^–1^), energy density *E*_d_ (Wh kg^–1^), and power density *P*_d_ (W kg^–1^) of the flexible device were calculated from the GCD curves by means of eqs 2–5:^35^
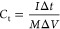
2
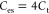
3
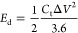
4
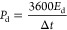
5where *M* is the total weight of the active material on both of the electrodes and Δ*V* is the operating voltage window after the *IR* drop.

## 3 Results and Discussion

### 3.1 Materials Characterization

In this study, high-SSA activated carbons were produced from corn derivatives (husk, fiber, grain, and cob) using a two-stage carbonization and potassium bicarbonate activation process. Using CHN elemental analysis, the compositions of the powdered dried corn derivatives were studied prior to the carbonization and activation process. The elemental compositions of the corn derivatives are given in Table 2. Elemental analysis revealed that the corn derivatives were mainly composed of 44–47% carbon, 43–49% oxygen, 5–7% hydrogen, and trace amounts of nitrogen (0.5–4%), with corn grain having 45.38% carbon and 0.85% nitrogen. Also, corn fiber contained the highest amount of nitrogen (3.72%) but the lowest amount of carbon (44.71%) among all of the samples. It is clear that the corn derivatives selected in this work mainly consist of carbon, nitrogen, and oxygen, which is desired for producing activated carbons.

**Table 2:** CHN Elemental Analysis Data for Corn Derivatives (Husk, Fiber, Grain, and Cob)

sample	% C (wt%)	% H (wt%)	% N (wt%)	% O (wt%)
corn husk	46.97	6.51	2.82	43.70
corn fiber	44.71	5.82	3.72	45.75
corn grain	45.38	5.89	0.85	47.88
corn cob	45.24	5.68	0.79	48.29

**Figure 1:**
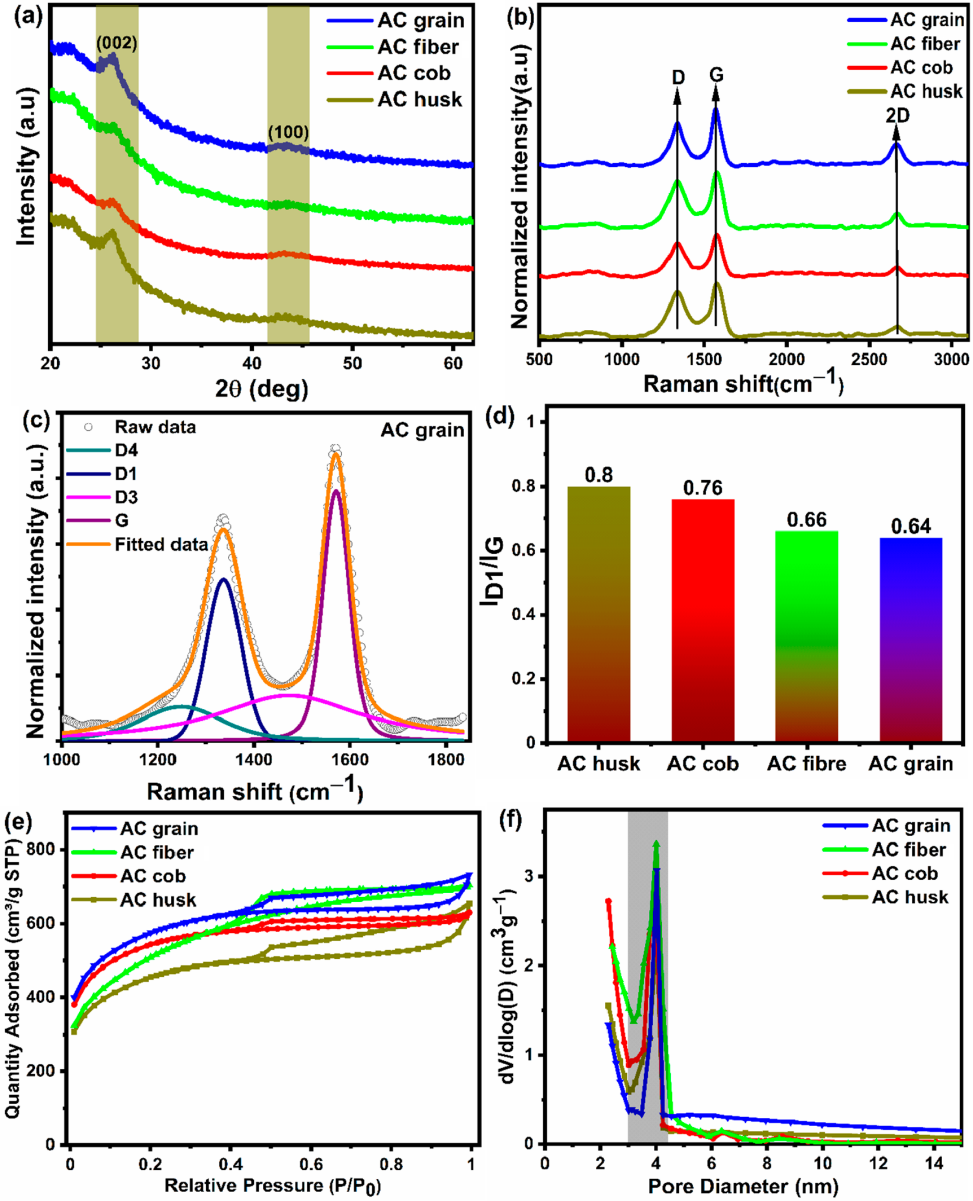
(a) XRD patterns of AC samples obtained from different corn parts. (b) Raman spectra of AC samples obtained from different corn parts. (c) Deconvoluted Raman spectra of AC grain sample, obtained by using Fityk software. (d) Degree of graphitization (*I*_D1_/*I*_G_) of AC samples obtained from different corn parts. (e) N_2_ adsorption/desorption isotherms. (f) PSD plot of AC samples obtained from different corn parts.

The structural and textural characteristics of the synthesized samples were analyzed via XRD, Raman spectra, and N_2_ adsorption isotherms. The XRD patterns of the AC samples obtained from different parts of corn are shown in Figure 1a. The XRD patterns of all the samples show two broad characteristic peaks at 2θ = ∼26° and ∼44°, corresponding to the (002) and (100) planes of predominantly amorphous carbon with partially graphitic structures.^8^ The graphitic plane (002) at ∼26° is responsible for the in-plane conductivity, which is particularly needed for electrochemical applications. Upon careful examination, the presence of one broader peak at ∼22.5°, corresponding to the (100) diffraction plane of the graphitic carbon embedded in amorphous carbon structures, can be observed.^36^ In order to further analyze the structures of the synthesized samples, Raman spectra were recorded, as shown in Figure 1b. All of the spectra consist of two prominent peaks centered at 1335 ± 5 and 1571 ± 4 cm^–1^, which are assigned to D and G bands, respectively. The G band represents the stretching vibration of ordered sp^2^ carbon, whereas the D band represents defective and disordered carbon structures.^37^ Additionally, all of the spectra exhibit a low-intensity peak at the higher wavenumber of 2666 ± 5 cm^–1^, corresponding to the 2D band, which reveals the presence of layered graphitic structures embedded inside the amorphous carbon framework.^38^ The D and G bands of all the activated carbon samples were further deconvoluted into four separate bands ascribed to the D4 band (polyenes/oligomers at 1210 cm^–1^), D1 band (disordered/defective carbon structure at 1336 cm^–1^), D3 band (amorphous carbon at 1485 cm^–1^) and G band (graphitic carbon at 1573 cm^–1^), as shown in Figures 1c and S1a–c.^39,40^ The relative intensity ratio of the D1 band to the G band (*I*_D1_/*I*_G_) is associated with the degree of graphitization in the synthesized samples, where the smaller the *I*_D1_/*I*_G_ ratio, the higher the degree of graphitization of the carbon material.^40,41^ As shown in Figure 1d, under the same preparation conditions, the AC grain sample exhibits the lowest *I*_D1_/*I*_G_ (0.64), which reflects the highest degree of graphitization or a higher number of sp^2^ hybridized graphitic carbons compared to the other samples.

To further understand the pore structure characteristics of the corn-based activated carbon samples, N_2_ adsorption/desorption studies were carried out. Figure 1e shows the obtained N_2_ adsorption/desorption isotherms, and Figure 1f shows the pore size distribution (PSD) plot of the as-prepared materials. Type IV isotherms with H4 hysteresis loops are visible for the AC husk and AC grain samples, whereas the AC fiber and AC cob samples display type I/IV isotherms with H4 hysteresis loops in the range of 0.4–0.9 *P*/*P*_0_, indicating the presence of hierarchical porous structures with dominant mesopores.^40,42^ The adsorption isotherms of all the samples display an uptake tendency in the lower *P*/*P*_0_ values (<0.4), indicating that the occurrence of a multilayer adsorption process takes place mainly in the mesoporous structures. Moreover, the adsorption/desorption isotherms of all the AC samples show a slight uptake in adsorption line at 0.9 < *P*/*P*_0_ < 1.0, which specifies the coexistence of meso- and macropores.^43^ From these adsorption/desorption isotherms, it can be concluded that the synthesized materials consist of a hierarchical porous structure with an interconnected mesoporous network. The SSA values of the corn-based activated carbon samples follow the order of AC grain (1805 m^2^ g^–1^) > AC fiber (1750 m^2^ g^–1^) > AC cob (1705 m^2^ g^–1^) > AC husk (1509 m^2^ g^–1^). These results indicate that a greater number of mesopores evolved in the case of the activated carbon obtained from corn grains (AC grain), as it had a high SSA of 1805 m^2^ g^–1^. This can be attributed to the carbonized corn grains being more effectively etched by the KHCO_3_ activating agent compared to the carbonized corn husk, fiber, or cob. Furthermore, the pore size distribution plot shown in Figure 1f indicates that all of the samples possessed mesoporous structures with the pores have narrow size range of 2–5 nm. A similar kind of behavior was observed by Hamouda et al.,^44^ Karnan et al.,^45^ and Shi et al.,^41^ who prepared high-SSA mesoporous carbons for supercapacitor applications. Shi et al.^41^ utilized bean curd stick byproducts to prepare high-SSA mesoporous carbon (2609 m^2^ g–1). Similarly, Hamouda et al.^44^ prepared mesoporous activated carbon from *Hibiscus sabdariffa* fruits (HBFs), which displayed a high surface area of 2609 m^2^ g^–1^ with the mesopores distributed in a size range of 2–5 nm. Hence, the BET and PSD findings reported in this work are consistent with similar kinds of findings reported in the literature.^41,44,45^Table 3 shows the pore characteristics of the activated carbons obtained from corn derivatives. According to Table 3 and the adsorption/desorption isotherm curves, all of the samples had mesoporous structures with an average pore size of 2–3 nm. However, the AC grain sample possessed a high SSA (1805 m^2^ g^–1^) and a higher SSA_meso_/SSA_T_ value (68.1%), which alludes to the existence of a larger number of mesopores in the as-synthesized sample. Materials with a high SSA_meso_/SSA_T_ ratio and an abundance of mesopores could be beneficial for enhanced electrolyte diffusion through the interlinked porous channels, yielding improved ion transfer rates and thereby increasing the electrochemical energy storage capabilities of the activated carbon obtained from corn grains. Therefore, it would be expected that the electrochemical performance of an AC grain-based electrode, especially the specific capacitance and capacity retention, would be significantly better than that for the other corn-based activated carbon samples.

**Table 3:** Characteristics of Pores in Corn-Based Activated Carbon Samples

sample ID	SSA_T_ (m^2^ g^–1^)	SSA_mic_ (m^2^ g^–1^)	SSA_meso_ (m^2^ g^–1^)	SSA_meso_/SSA_T_ (%)	*V*_T_ (cm^3^ g^–1^)	*V*_mic_ (cm^3^ g^–1^)	*V*_meso_ (cm^3^ g^–1^)	*V*_meso_/*V*_T_ (%)	pore size (nm)
AC husk	1508	643	1067	57.3	1.01	0.48	0.53	52.4	2.2
AC cob	1770	695	1075	60.7	0.97	0.36	0.61	61.8	2.3
AC fiber	1749	584	1165	66.6	1.08	0.35	0.73	67.5	2.8
AC grain	1804	574	1230	68.1	1.07	0.31	0.76	71.1	2.6

**Figure 2:**
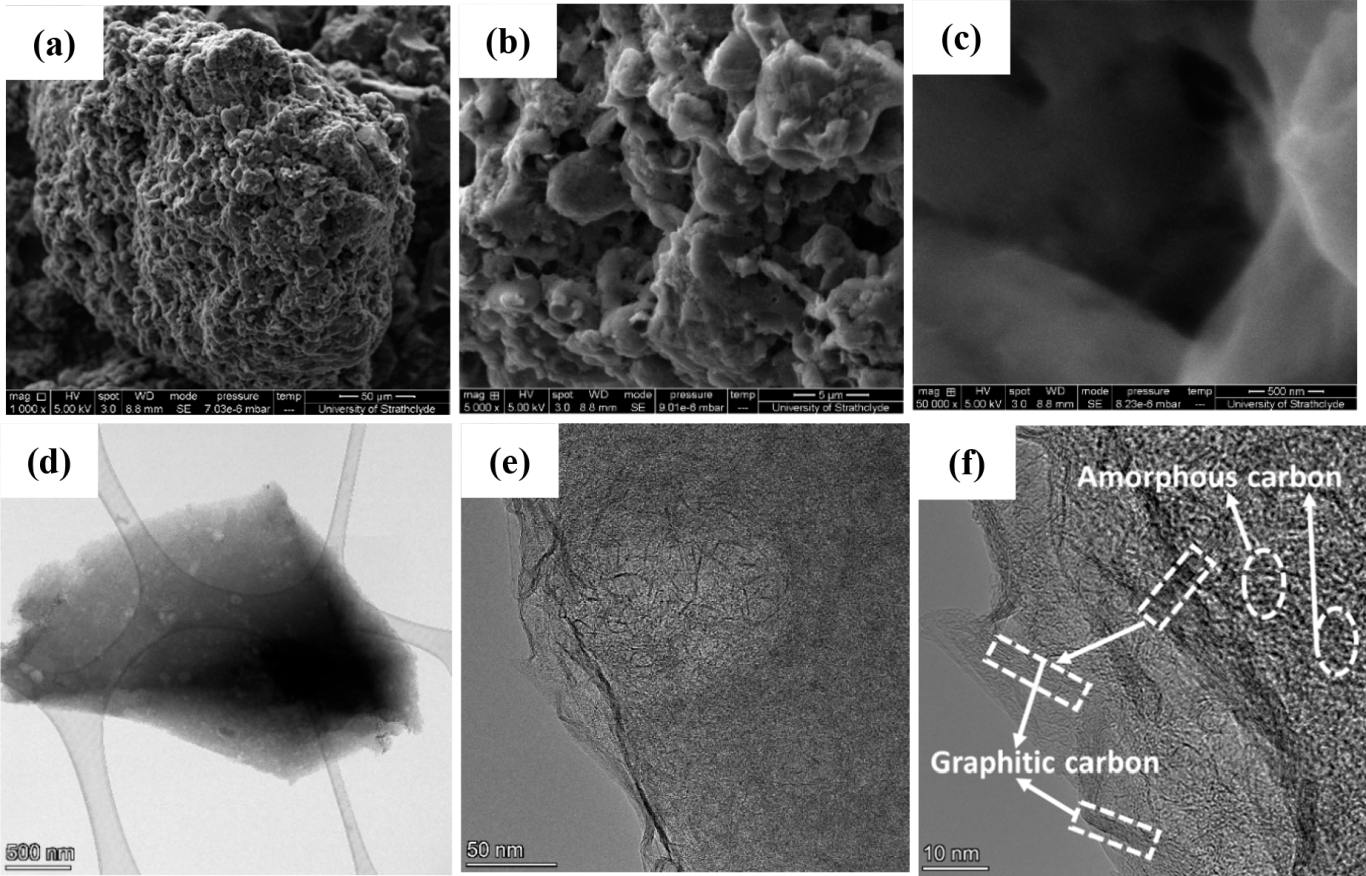
(a–c) FESEM images and (d–f) HRTEM images of the AC grain sample at different magnifications.

Next, the morphologies of the corn-based activated carbons were studied by FESEM. Figure 2a–c shows FESEM images of the activated carbon obtained from corn grains; FESEM images of the remaining corn derivatives are shown in the Supporting Information, Figure S2. The FESEM images of all the corn-based activated carbon samples display disordered and uneven surface morphologies with aggregation of the porous carbons stacked with many graphitic layers. However, the FESEM image of the AC grain sample revealed a much higher porosity, with an uneven and rough surface containing numerous small pores of various sizes and shapes. We believe that the strong etching induced by the KHCO_3_ activation resulted in the uneven corrosion of the carbon surface, thereby causing the formation of hierarchical porous structures within the small number of graphitic layers that were present on the interior/exterior surface of the porous amorphous structure. HRTEM images of AC grain are shown in Figure 2d–f, and HRTEM images of the remaining corn derivatives are shown in Figure S3. It can be observed that the AC grain sample has many defects, and it exhibits an amorphous carbon structure with numerous pores, as shown in Figure 2d. The presence of graphitic carbon layers embedded in the porous carbons is verified from the high-magnification HRTEM images shown in Figure 2e,f. The high-magnification TEM image (Figure 2f) shows the presence of an amorphous carbon structure connected with graphitic carbon layers. It has been reported that this kind of porous structure is extremely important for supercapacitor applications because it provides electroactive sites to trap and adsorb electrolyte ions, while the graphitic layers provide interconnected transport pathways, which improves the mobility of ions into deeper sections of the porous network.^46,47^

**Figure 3:**
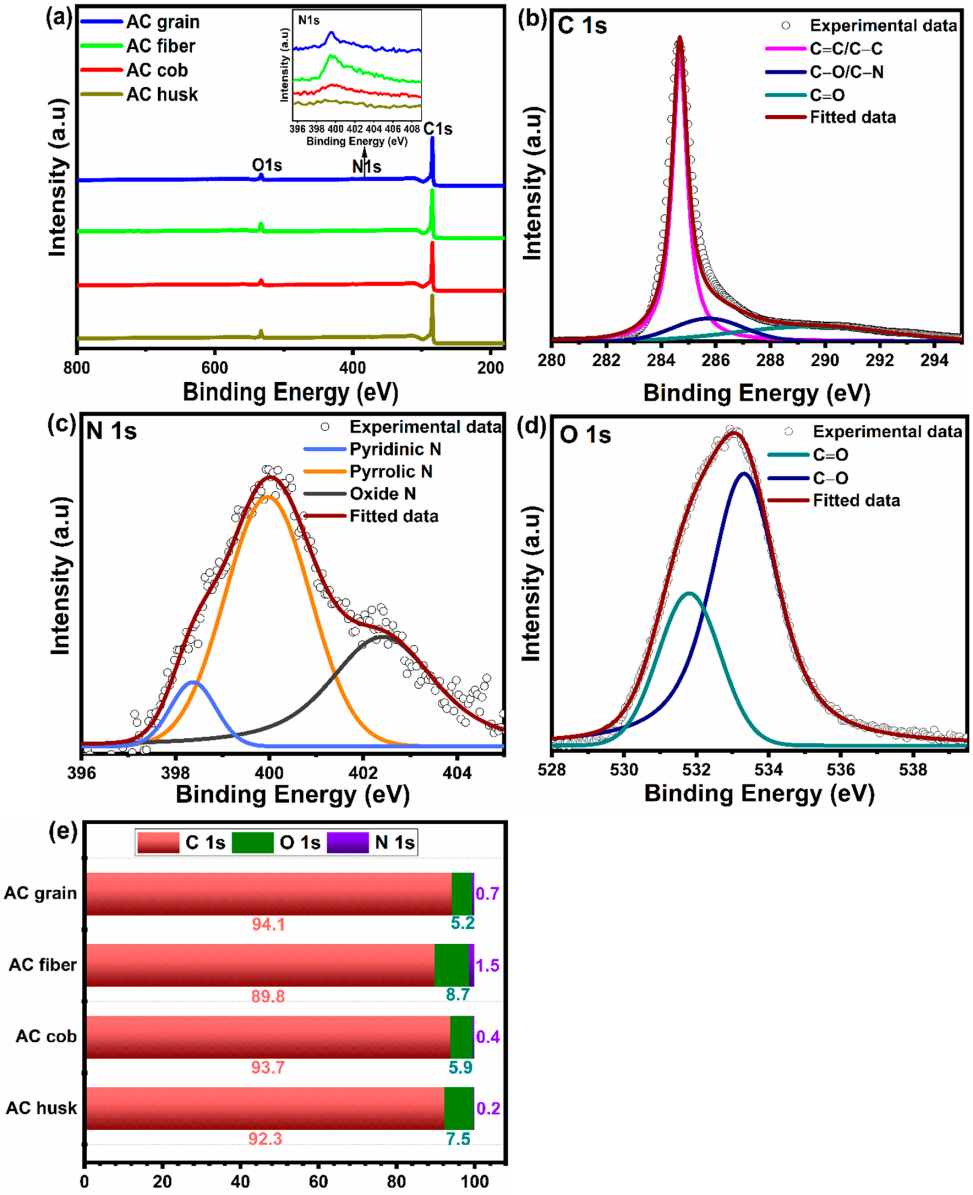
(a) XPS survey spectra of the corn-based activated carbon samples, and deconvoluted XPS core-level spectra of (b) C 1s, (c) N 1s, and (d) O 1s of the AC grain sample. (e) Plot showing the relative wt % of different elements present in the corn-based activated carbon samples based on XPS peak analysis.

XPS measurements were also used to identify the elemental compositions of the corn-based activated carbon materials. The overall XPS survey scans of the corn-based activated carbons in Figure 3a mainly comprise a high-intensity C 1s peak at 284.5 eV and low-intensity O 1s and N 1s (shown in the inset of Figure 3a) peaks at 532.5 and 400 eV, respectively. The XPS studies show that carbon is the predominant element in all of the corn-based activated carbons with small amounts of oxygen and nitrogen also present, most likely on the surface of the carbon framework. Peak deconvolution was carried out to determine the type of chemical bonds present in the corn-based activated carbon samples, and the deconvoluted spectra of the individual elements are displayed in Figures 3b–d and S4a–i. The C 1s spectra was deconvoluted into three peaks, as illustrated in Figure 3b, where the peaks at 284.5, 286, and 289 eV correspond to C=C/C–C, C–O/C–N, and C=O respectively.^41,48^ The deconvoluted N 1s spectrum of AC grain shown in Figure 3c indicates the existence of pyridinic N, pyrrolic N, and N oxide at 398.4, 400 ± 0.5, and 402.7 eV, respectively.^41,49^ According to the literature, pyridinic N groups serve as electrochemically active sites to enhance the specific capacitance, whereas pyrrolic N improves the ion transfer rate from the electrolyte to the electrode, and N oxide promotes redox reactions to increase pseudocapacitance.^50−52^ Hence, it can be concluded that these N-containing functional groups improve the electrochemical properties of activated carbon to a significant extent.^53^ The high-resolution O 1s spectrum of AC grain is also well deconvoluted into two peaks located at 531.6 and 533.4 eV, which represent C=O and C–O, respectively, as shown in Figure 3d.^20,41^ These oxygen functional groups are crucial in enhancing the wettability of the activated carbon, increasing the access to electrolyte ions in order to completely utilize the high specific surface area and improve the electrochemical energy storage.^48^ The elemental composition of C, N, and O in the samples was obtained from the core energy levels of the individual elements by using the peak areas. The peak area (*I*_i_) of each element was divided by its sensitivity factor (*S*_i_) to obtain a normalized peak area, *I*_i_/*S*_i_. The elemental composition (*X*_i_) of each element was then calculated using the normalized peak area of each element divided by the sum of all the normalized peak areas.^54^ From Figure 3e, it can be clearly observed that the relative amount of carbon varied from 89.8 to 94.1% in all the samples with AC grain composed of 94.1% carbon, 5.2% oxygen, and 0.7% nitrogen. Among all the samples, AC fiber contained the highest amount of nitrogen (1.5%) and the lowest amount of carbon (89.8%). Lastly, contact angle measurements were evaluated to elucidate the electrolyte wettability of the electrode surface.^34^Figure S5 shows the images of a 1 M Na_2_SO_4_ droplet in contact with the corn-based activated carbon electrode surfaces, where the AC fiber electrode showed the lowest contact angle of 70.5° and the AC grain, AC cob, and AC husk electrodes displayed electrolyte contact angles of 72.3, 84.3, and 88.8°, respectively. The lower contact angles of the AC grain and AC fiber electrodes can be credited to their high SSAs and higher amounts of N and O heteroatoms. These characteristics result in an improved affinity toward the 1 M Na_2_SO_4_ electrolyte, thereby leading to a lower equivalent resistance. Although AC cob and AC husk possess higher amounts of oxygen, their contact angles are higher than the contact angle of AC grain. This may be attributed them having a lower SSA and degree of graphitization compared to the AC grain sample. The lower contact angle, higher SSA, and higher degree of graphitization of the AC grain electrode results in improved wettability, higher charge accumulation, and enhanced ion transportation inside the porous medium. These contact angle findings are in correlation with the Raman, XPS, and N_2_ adsorption–desorption results discussed earlier.

### 3.2 Electrochemical Analysis of the Electrodes

**Figure 4:**
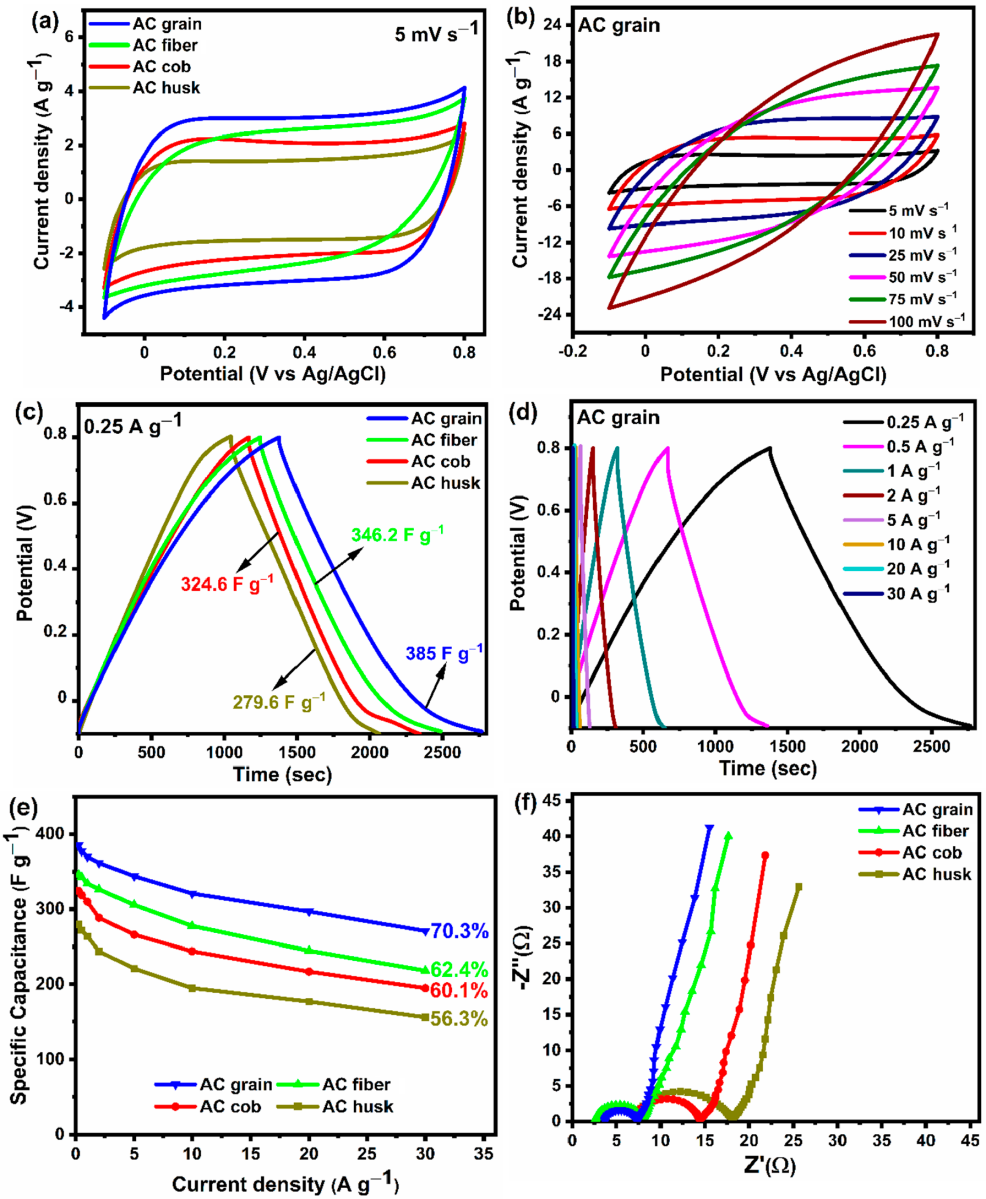
(a) CV curves of the corn-based activated carbons at a scan rate of 5 mV s^–1^. (b) CV curves of AC grain at various scan rates. (c) GCD curves of the corn-based activated carbon samples at a current density of 0.25 A g^–1^. (d) GCD curves of AC grain at various current densities. (e) Specific capacitance vs current density of the corn-based activated carbons. (f) Nyquist plots of the corn-based activated carbons tested in a 1 M Na_2_SO_4_ electrolyte in a three-electrode configuration.

In order to study the electrochemical behavior of the corn-based activated carbon electrodes, electrochemical tests such as CV, GCD, and EIS were performed initially by using a 1 M Na_2_SO_4_ electrolyte in a three-electrode configuration within a 0.9 V potential window. As shown in Figure 4a, the CV curves of the activated carbons corresponding to grain, husk, and cob exhibit ideal “rectangular”-shaped curves at a scan rate of 5 mV s^–1^. In contrast, although AC fiber displayed similar current densities, its CV curve did not display the classic rectangular shape, indicating that the capacitive contribution of AC fiber is not as ideal as for that of the other samples. This might be due to the increased amount of oxygen and nitrogen functional groups on the surface of this activated carbon, which promote faradaic reactions, resulting in pseudocapacitance.^52,55^ Nevertheless, these (quasi-)rectangular-shaped CV curves indicate that all of the corn-based activated carbon samples display an electrochemical double-layer-type charge storage mechanism. The lower SSA of the AC husk sample resulted in a smaller rectangular curve with a low current density, which indicates a lower specific capacitance or lower energy storage. It can be observed from Figure 4a that with the same 1 M Na_2_SO_4_ electrolyte and scan rate (5 mV s^–1^), AC grain demonstrates a greater capacitance, as its CV curve has a significantly larger area compared to that of the activated carbon from cob, husk, and fiber. This can be understood from the fact that AC grain possesses a higher SSA and a more porous structure, which resulted in an increased amount of surface-active sites for the electrolyte ions to access. Figure 4b shows the CV curves of the AC grain working electrode tested at different scan rates from 5 to 100 mV s^–1^. It can be seen that the CV curves resemble rectangular shapes at lower scan rates (5–25 mV s^–1^) and quasi-rectangular-like shapes at higher scan rates (50–100 mV s^–1^) within the same potential window, indicating the superior adsorption and desorption of electrolyte ions on the electrode surface at lower scan rates (5–25 mV s^–1^). The reason for the quasi-rectangular-shaped curves at higher scan rates (50–100 mV s^–1^) is the limited reaction time for electrolyte motion inside the pores of the electrode, leading to a change in its double-layer characteristics.^56^ More specifically, the limited reaction time at higher scan rates prevents ionic movement inside the pores of the electroactive material, leaving only the exterior surface of the material available for charge storage. In contrast, the electrolyte ions can access the entire surface area of the electrode material at lower scan rates. The specific capacitance and rate capability of the corn-based activated carbon samples was evaluated from the GCD curves (Figure 4c). The specific capacitance values were calculated using eq 1. At a current density of 0.25 A g^–1^, all the samples displayed an isosceles triangular-shaped charge–discharge behavior, which further confirms their double-layer charge storage performance. It is very clear from Figure 4c that AC grain exhibited the longest discharge time, followed by AC fiber, AC cob, and AC husk. This is the same trend revealed by the CV curves in Figure 4a. The AC grain sample possessed a remarkable specific capacitance (*C*_s_) of 385 F g^–1^ at 0.25 A g^–1^, followed by AC fiber (346.2 F g^–1^), AC cob (324.6 F g^–1^), and AC husk (279.6 F g^–1^). Figure 4d shows the corresponding GCD curves of AC grain at various current densities ranging from 0.25 to 30 A g^–1^. All of the curves have a shape similar to an isosceles triangle, further demonstrating the superior electrochemical reversibility and rate capability of the AC grain electrode at higher current densities. A specific capacitance of 271 F g^–1^ could still be attained at the high current density of 30 A g^–1^, which indicates the attractive rate capability of the activated carbon obtained from corn grains (AC grain). The reduced *C*_s_ values at higher current densities can be related to the reduced diffusion time for the transport of Na^+^/SO_4_^2–^ ions from the electrolyte to the surface-active sites of the electrode. Figure 4e displays the specific capacitance of the corn-based activated carbon samples as a function of current density, along with their capacitance retention capabilities. From Figure 4e, it is clear that AC husk retains 56.3% of its capacitance even at the high current density of 30 A g^–1^, which is the lowest among all the samples. In contrast, AC grain displays an excellent capacitance retention of 70.3%, which can be attributed to its hierarchical porous structure, high SSA, high degree of graphitization, and interconnected porous network. The high SSA of AC grain generates a greater number of surface-active regions for the ions to access, while its high degree of graphitization with its interconnected porous network provides efficient ion transfer/diffusion channels and promotes electrolyte ion penetration into deeper regions of the porous electrode framework. Figure 4f shows the Nyquist plots of the corn-based activated carbon samples measured within a frequency range of 0.01 mHz to 100 kHz with an AC perturbation of 5 mV. The Nyquist plots of all the electrodes consist of a straight line in the low-frequency region and a semicircle in the high-frequency region.^57^ The diameter of the semicircle in the high-frequency region gives the charge transfer resistance (*R*_ct_), which arises from the ionic resistance inside the pores of the electroactive material. In the presence of a 1 M Na_2_SO_4_ electrolyte, *R*_ct_ follows the order of AC grain (3.6 Ω) < AC fiber (5.2 Ω) < AC cob (7.25 Ω) < AC husk (10.7 Ω). The equivalent series resistance (*R*_s_), which combines the internal resistance of the current collector, the resistance of the electrolyte ions, and the contact resistance of the electroactive material with the collector, is represented by the intercept of the semicircle on the *x*-axis in the high-frequency region. The *R*_s_ values of AC husk, AC cob, AC fiber, and AC grain are 7.3, 7.15, 2.58, and 3.7 Ω respectively. AC fiber has the lowest *R*_s_ value out of all the samples, which could be attributed to its high oxygen and nitrogen content, which increases the surface wettability of the electroactive material with the current collector, thereby reducing the contact resistance. To better study the electrochemical properties of the samples, we fitted the impedance spectra of all the corn-based activated carbon electrodes, and the corresponding equivalent circuits are shown in the Supporting Information (Figure S6). The equivalent circuits consist of a combination of the following components: *R*_s_, *R*_ct_, the double-layer capacitance (*C*_dl_), the constant phase element (CPE), and the Warburg impedance related to the diffusion of electrolyte ions (*W*). The *R*_ct_–*C*_dl_ circuit is responsible for the ion transfer inside the pores of the electroactive material. The almost inclined line that is observed in the low-frequency region in the Nyquist plots indicates the capacitive behavior of the corn-based activated carbon electrodes, which is represented by CPE in the equivalent circuits.^34^ The Nyquist data suggest that the electrode material based on corn grain-derived activated carbon has excellent charge transfer properties. From the above results, the superior electrochemical features of AC grain can be summarized as follows: (1) its high SSA offers sufficient surface-active regions for the adsorption of charge carriers, leading to high specific capacitance. (2) Its numerous transport channels and shorter diffusion paths, supplied by its hierarchical porous structure and higher degree of graphitization, are helpful for increasing its rate performance and decreasing *R*_ct_. (3) Its high carbon content and suitable amount of N and O functional groups create additional pseudocapacitance, which increases its specific capacitance to some extent.

**Figure 5:**
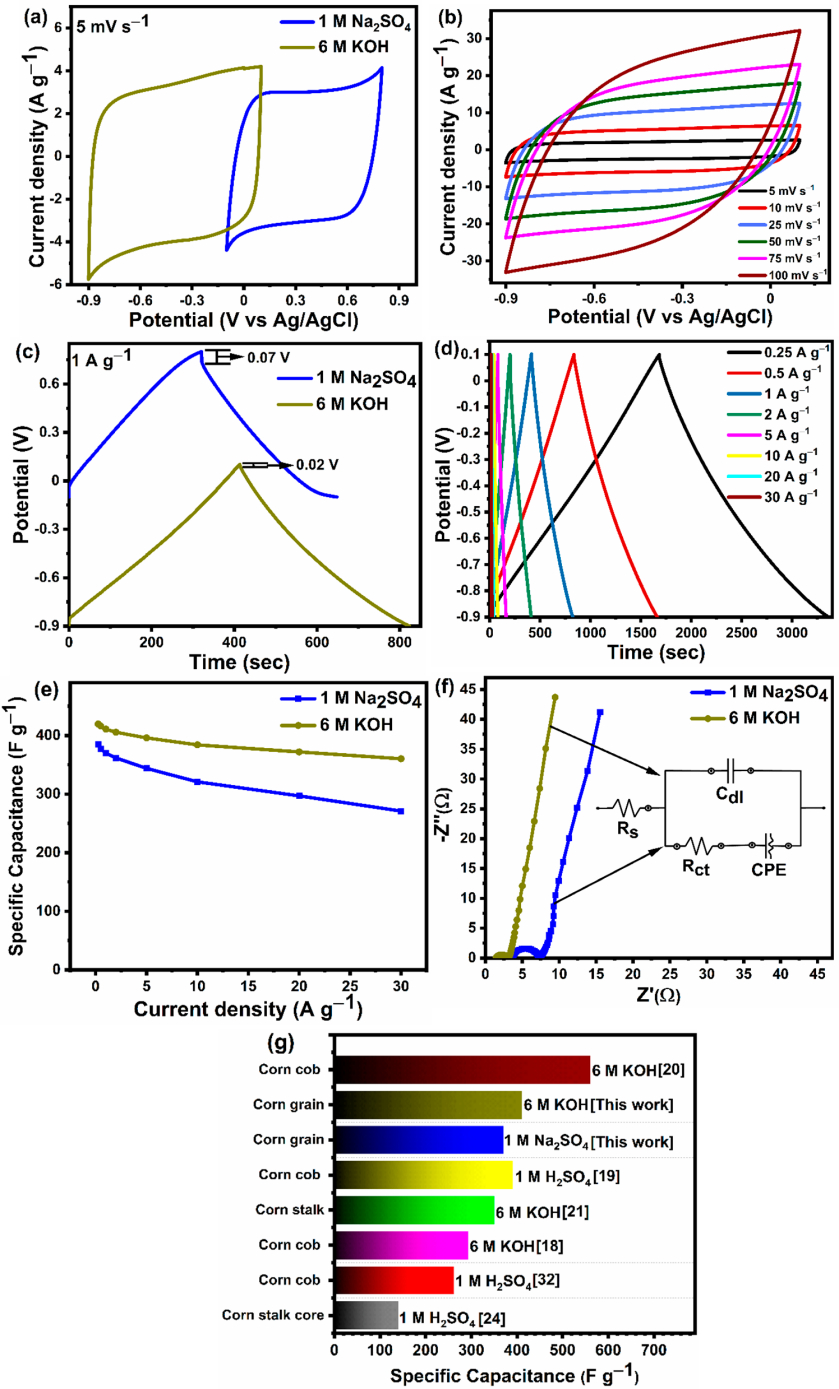
Electrochemical performance of the AC grain electrode. (a) CV curves of the AC grain electrode at a scan rate of 5 mV s^–1^ in two different electrolytes. (b) CV curves of the AC grain electrode at different scan rates in the 6 M KOH electrolyte. (c) GCD curves of the AC grain electrode at a current density of 1 A g^–1^ in the two different electrolytes. (d) GCD curves of AC grain at different current densities in the 6 M KOH electrolyte. (e) Specific capacitance plotted as a function of current density of the AC grain sample in two different electrolytes. (f) Nyquist plot of the AC grain electrode tested in two different electrolytes. (g) Plot showing a comparison of the electrochemical performance of the AC grain electrode with that of other activated carbons reported in the literature.

In order to determine the impact of the electrolyte type on the electrochemical performance, the electrochemical tests were repeated using a 6 M KOH electrolyte. It is important to note that one essential criterion that must be established for a supercapacitor system to function effectively is the electrolyte type. Figure 5a displays the CV curves of the AC grain electrode tested in the 1 M Na_2_SO_4_ and 6 M KOH aqueous electrolytes at a scane rate of 5 mV s^–1^. It can be seen that the AC grain electrode showed a classical rectangular-shaped CV curve in both the 6 M KOH and 1 M Na_2_SO_4_ electrolytes, suggesting it has a double-layer charge storage mechanism with excellent adsorption and desorption of ions in both media.^58^ However, the current response and the area occupied by the CV curve of the AC grain electrode in the 6 M KOH electrolyte are greater compared to those in the 1 M Na_2_SO_4_ electrolyte, indicating a better electrochemical performance. Figure 5b shows the CV curves of the AC grain electrode at various scan rates in the 6 M KOH electrolyte. It can be seen that the current response starts to increase with an increasing scan rate while still retaining a rectangular shape, indicating the strong rate capability of the AC grain in the 6 M KOH electrolyte. Figure 5c depicts typical GCD curves of the AC grain electrode in 1 M Na_2_SO_4_ and 6 M KOH electrolytes at a current density of 1 A g^–1^. The appearance of a triangular-shaped charge–discharge curve in the two electrolytes implies that the charge storage is predominantly a double-layer type. However, the charge–discharge time of the AC grain electrode in the 6 M KOH electrolyte is slightly higher than in the 1 M Na_2_SO_4_ electrolyte, which indicates that a higher specific capacitance can be achieved in the 6 M KOH electrolyte. Moreover, a smaller *IR* drop of 0.02 V can be observed in the GCD curves at 1 A g^–1^ in 6 M KOH, indicating a lower *R*_s_ value in the 6 M KOH electrolyte. These GCD curves are again consistent with the CV curves shown in Figure 5a. The specific capacitance values of the AC grain electrode, which were calculated from eq 1 for the two electrolytes at 1 A g^–1^, are ranked as follows: 6 M KOH electrolyte (411 F g^–1^) > 1 M Na_2_SO_4_ electrolyte (370 F g^–1^). A GCD study with current densities ranging from 0.25 to 30 A g^–1^ was conducted for the AC grain electrode in the 6 M KOH electrolyte, as illustrated in Figure 5d. It is clear that a symmetric triangular-shaped curve was maintained even at higher current densities, indicating the excellent rate capability of the electrode in the 6 M KOH electrolyte. With an increase in the current density from 0.25 to 30 A g^–1^, the capacitance retention of the AC grain electrode was 73.2% and 85.7% of its lowest rate capacitance in the 1 M Na_2_SO_4_ and 6 M KOH electrodes, respectively (Figure 5e). This high capacitance retention of the AC grain electrode at the high charge–discharge rate of 30 A g^–1^ demonstrates its superior rate capability, which can be attributed to the larger SSA and interconnected porous framework of AC grain. Furthermore, the increased capacitance retention of the AC grain electrode in the 6 M KOH electrolyte shows that the dissolved K^+^ and OH^–^ ions have faster ionic mobilities under higher current densities during the charge–discharge process.^58^ Using EIS, the charge transport kinetics of the AC grain electrode was further investigated in the two electrolytes within a frequency range from 0.01 to 100 kHz. As shown in Figure 5f, the *R*_s_ value of the AC grain electrode is 3.70 and 1.39 Ω in the 1 M Na_2_SO_4_ and 6 M KOH electrolytes, respectively. As the same AC grain electrode was employed in the two electrolytes, the difference in the *R*_s_ value is mostly due to the ionic conductivity of the electrolyte and, to some extent, the current collector. We believe that the relatively lower *IR* drop (Figure 5c) was what was primarily responsible for the lower *R*_s_ values in the 6 M KOH and 1 M Na_2_SO_4_ electrolytes. The estimated *R*_ct_ value of the AC grain electrode was 1.72 Ω in the 6 M KOH electrolyte and 3.57 Ω in the 1 M Na_2_SO_4_ electrolyte, conveying that there was a good charge transfer rate for the Faradaic reaction involved in the pseudocapacitance process. Notably, the equivalent circuit of the AC grain electrode in the 1 M Na_2_SO_4_ and 6 M KOH electrolytes includes the *R*_s_, *R*_ct_, *C*_dl_, and CPE elements, as shown in the inset of Figure 5f. Figure 5g shows a comparison of the specific capacitance of the AC grain electrode determined in this work with that of activated carbons obtained from different corn samples reported in the literature in three different electrolytes (Na_2_SO_4_, KOH, and H_2_SO_4_).^18−21,24,32^Figure 5g clearly illustrates that the performance of the AC grain electrode studied in this work is comparable and even better than some of the previously reported results of other corn-based activated carbons. From the above results, we can conclude that the AC grain electrode achieved an outstanding electrochemical performance with a higher specific capacitance, superior rate capability, and better electronic conductivity, along with lower series and charge transfer resistances, in the 6 M KOH aqueous electrolyte.

### 3.3 Flexible Supercapacitor Performance

**Figure 6:**
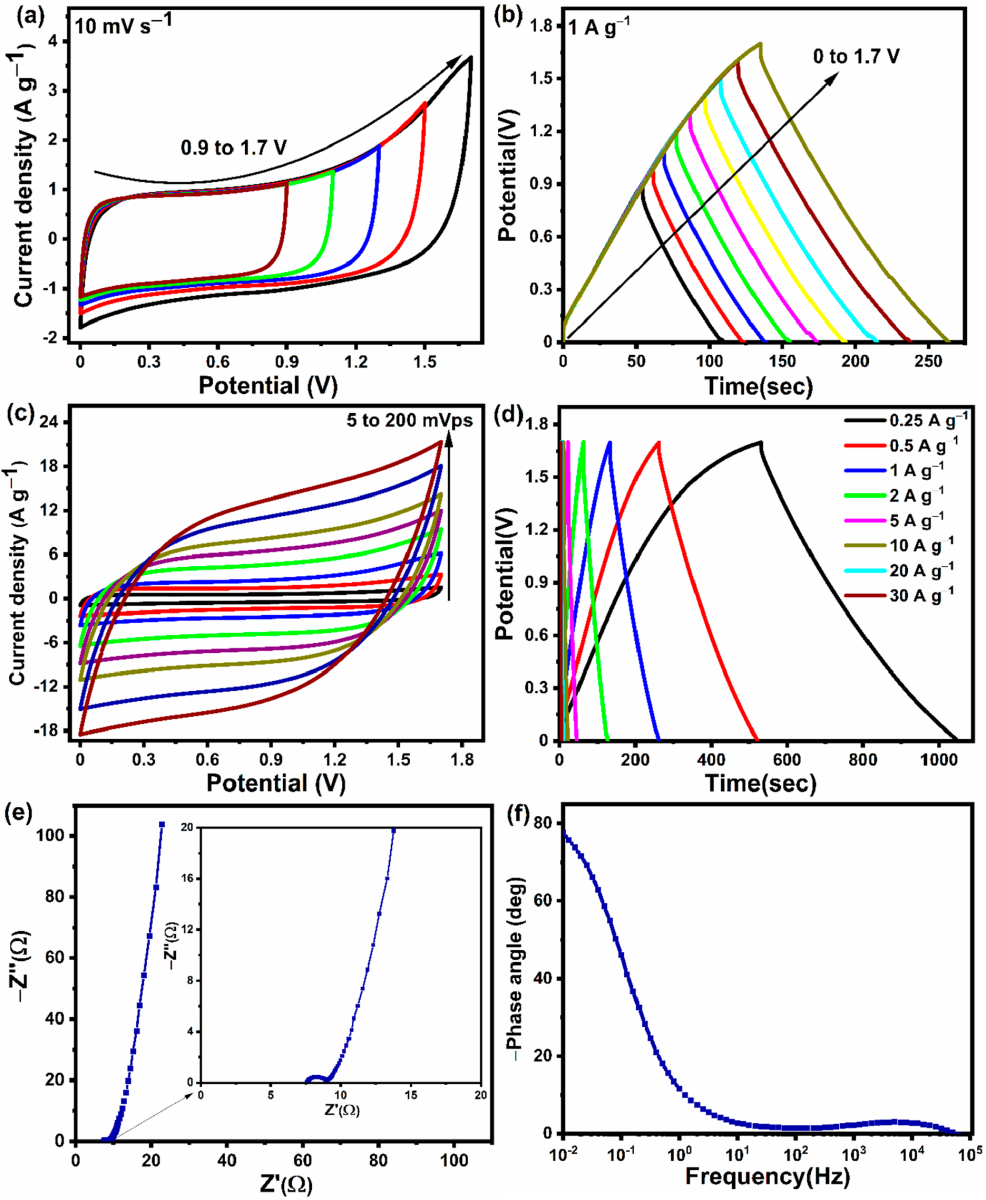
(a) CV curves of the flexible supercapacitor prepared with the AC grain sample and HEC/KOH electrolyte measured at 10 mV s^–1^ over different voltage ranges. (b) GCD curves measured at 1 A g^–1^ over different potential ranges. (c) CV curves measured at various scan rates. (d) GCD curves recorded at various current densities. (e) EIS plot and (f) phase plot of the flexible supercapacitor prepared with the AC grain sample and HEC/KOH electrolyte.

To further demonstrate the application of the AC grain material in flexible electrochemical supercapacitors, two AC grain electrodes, coated with a HEC/KOH gel electrolyte using a slot-die coater, were assembled into a symmetrical supercapacitor. As shown in Figure 6a, the CV tests that were performed at a scan rate of 10 mV s^–1^ and a voltage increment of 0.2 V in the range from 0.9 to 1.7 V did not disclose any substantial increase in anodic current density even at 1.7 V, which indicates that the polymer gel electrolyte was not being decomposed. However, beyond 1.7 V, the anodic current density increased significantly, revealing the decomposition of the biopolymer electrolyte (as shown in Figure S7a). These results also indicate that the flexible symmetric supercapacitor could be charged/discharged reversibly and was stable within the 1.7 V potential window. However, such a qualitative assumption can only be validated by quantitative techniques like GCD. The charge–discharge tests at a current density of 1 A g^–1^ (shown in Figure 6b) within the 1.7 V potential window did not reveal any obvious plateau, which indicates that the electrolyte was stable and did not decompose. As a result, the enlarged potential window of 0–1.7 V was chosen as the stable working voltage range for subsequent electrochemical tests. Additional CV tests were performed by varying the scan rate from 5 to 200 mV s^–1^, as shown in Figure 6c. Even at higher scan rates, the CV curves of the AC grain-based flexible supercapacitor retained their rectangular shape, indicating the optimal double-layer behavior and superior rate performance of the symmetric supercapacitor. Interestingly, no obvious redox peak is visible from the CV curve of the device, which indicates the charge storage mechanism in the HEC/KOH gel electrolyte is purely of a double-layer type. The GCD profiles illustrated in Figure 6d show a linear and symmetrical triangular-shaped charge–discharge behavior, validating the pure double-layer charge storage mechanism in the flexible supercapacitor, which is consistent with the CV results discussed above. Using eqs 2–5, the specific capacitance, energy density, and power density of the flexible supercapacitor were evaluated from the discharge curves of the GCD data. At the current densities of 0.25, 0.5, 1, 2, 5, 10, 20, and 30 A g^–1^, the AC grain-based flexible supercapacitor showed *C*_s_ values of 77.16, 76.4, 75.2, 72.9, 67.6, 62.2, 56.1, and 50.4 F g^–1^, respectively. When the current density was increased from 0.25 to 30 A g^–1^, the supercapacitor still retained 65.3% of its initial *C*_s_ at 0.25 A g^–1^, revealing the excellent rate capability of the device. The EIS plot of the device (Figure 6e) shows an initial series resistance (*R*_s_), a semicircle in the high-frequency region that is ascribed to the charge transfer resistance (*R*_ct_) originating from a distributed Faradaic reaction within the structure, and an almost vertical line at low frequencies, indicating a near-ideal capacitive-type storage mechanism. By fitting the EIS curve of the flexible supercapacitor, the *R*_s_ and *R*_ct_ values were found to be 7.38 and 1.58 Ω, respectively. The optimal capacitive behavior and minimal electrolyte ion diffusion resistance of the material are indicated by the almost vertical line characteristic seen in the low-frequency region. Figure 6f displays the frequency dependent phase angle (Bode plot) of the flexible supercapacitor. The flexible supercapacitor displayed a phase angle of −77.8° in the low-frequency region, which is close to the phase angle of an ideal supercapacitor (−90°). Therefore, it is clear that the flexible supercapacitor displayed an excellent capacitive performance with a lower charge transfer resistance and higher phase angle, which can be ascribed to the combined effect of the high specific surface area, high degree of graphitization, and high carbon content of the activated carbon obtained from the corn grains.

**Figure 7:**
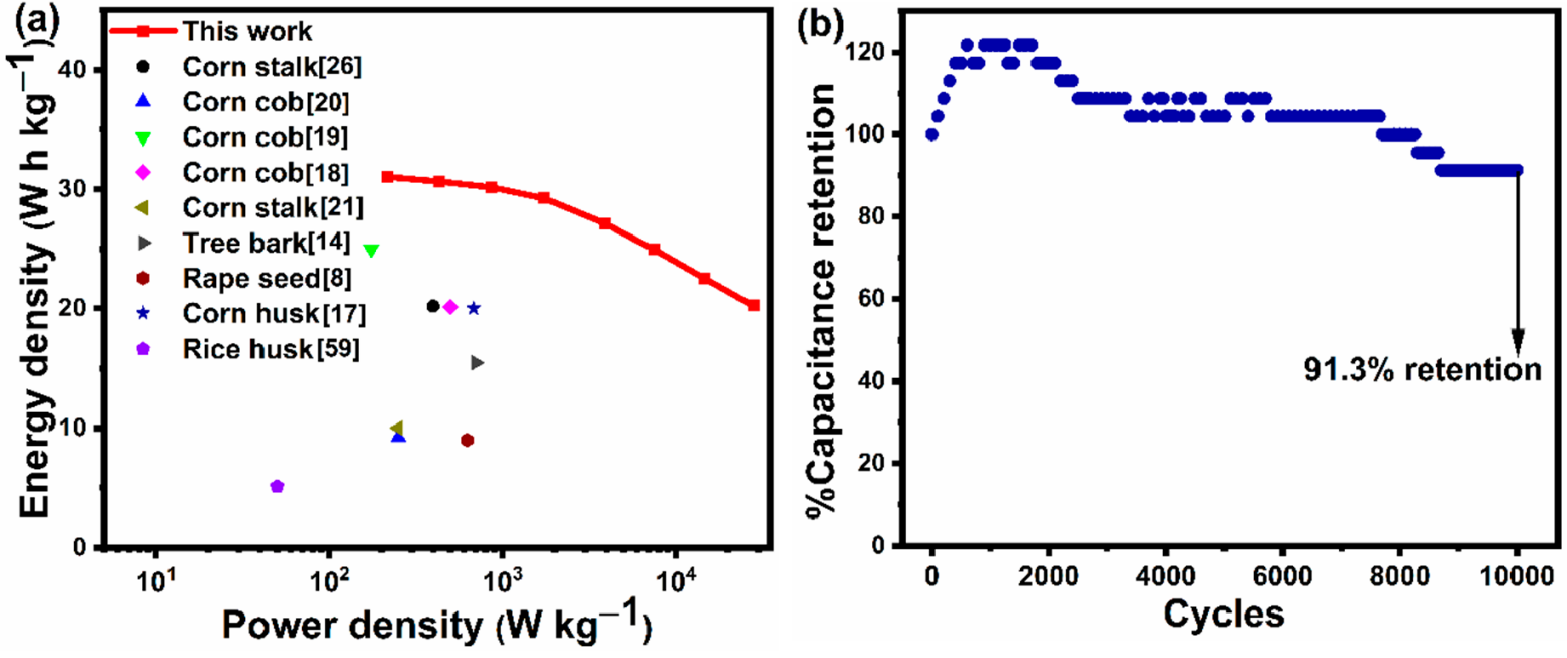
(a) Ragone plot of the AC grain-based flexible supercapacitor compared to other supercapacitors fabricated using other biomass-based ACs reported in the literature. (b) Cyclic stability of the AC grain-based flexible supercapacitor performed over 10 000 cycles at a current density of 5 A g^–1^.

Figure 7a shows the Ragone profile of the flexible supercapitor created with AC grain electrodes and the HEC/KOH gel polymer electrolyte, which was plotted using the *E*_d_ and *P*_d_ values calculated using eqs 4 and 5, in comparison to that reported in the literature for other corn-based ACs. The flexible supercapacitor prepared using AC grain attained an outstanding *E*_d_ of 31.1 Wh kg^–1^ at a *P*_d_ of 215 W kg^–1^, and it can be observed from the curve in Figure 7a that when *P*_d_ reached 28.0 kW kg^–1^, the *E*_d_ was still 20.2 Wh kg^–1^. Thus, the energy and power density values are comparable or even better than those of previously reported corn-based (or other biomass-derived) porous carbonaceous supercapacitors.^8,14,17−21,26,59^ For example, Yue et al.^21^ reported the fabrication of a coin cell-type supercapacitor with high-surface-area (2152 m^2^ g^–1^) activated carbon from corn stalk, which obtained an energy density of 10.01 Wh kg^–1^ in a 1 M Na_2_SO_4_ electrolyte under a 1 V potential window. Similarly, Wang et al.^20^ fabricated an aqueous symmetric supercapacitor with high-surface-area (2508 m^2^ g^–1^) corn cob-derived activated carbon and a 6 M KOH electrolyte, which exhibited an energy density of 9.24 Wh kg^–1^. In both of these cases, it can be observed that the energy density values are far lower than those in the present work, even though these corn-based activated carbons possessed higher surface areas (>2152 m^2^ g^–1^) than the corn-based activated carbon in this work (1804.90 m^2^ g^–1^). This can be attributed to the lower operating potential window (1 V in the first two cases described), which is lower than the operating potential window of 1.7 V used in the current study. It is clear from eq 4 that the potential window is directly proportional to the energy density of the supercapacitor. From the Ragone plot, it can also be observed that the flexible supercapacitor created with AC grain electrodes and the HEC/KOH gel polymer electrolyte displayed a higher electrochemical performance than the devices reported by Karnan et al.^19^ and Rani et al.^17^, who employed ionic liquid and organic electrolytes, respectively. Karnan et al.^19^ used the EMIMBF_4_ ionic liquid electrolyte to fabricate a Swagelok cell-type supercapacitor with corn cob-based activated carbon (SSA of 800 m^2^ g^–1^). Similarly, Rani et al.^17^ employed a 1 M TEABF_4_/AN organic electrolyte to fabricate a supercapacitor with corn husk-based activated carbon (SSA of 1370 m^2^ g^–1^). We believe that the lower SSAs of the corn cob-^19^ and corn husk-based^17^ activated carbons in the aforementioned cases had an impact on the *E*_d_ of the supercapacitors when compared to the results of the current investigation. Furthermore, as shown in Figure 7a, the flexible supercapacitor created with AC grain electrodes and the HEC/KOH gel polymer electrolyte exhibited a better electrochemical performance compared to the supercapacitors fabricated from tree bark-,^14^ rapeseed meal-,^8^ and rice husk-based^59^ activated carbons reported in the literature.

Next, the electrochemical cyclic retention ability of the flexible supercapacitor was analyzed under continuous charge–discharge cycles for 10 000 cycles at a current density of 5 A g^–1^, as shown in Figure 7b. It is evident that the specific capacitance initially increased during the first few 1000 cycles, as the percentage of the capacitance that was retained increased by more than 20% of its initial value. This rise in the percentage capacitance retention in the initial few cycles can be attributed to the complete utilization of the electrode framework during the continuous adsorption/desorption of the electrolyte ions. After this, there was a steady decrease in the capacitance, with 8.7% of initial capacitance loss at the end of 10 000 cycles. The specific capacitance may gradually decrease for a variety of reasons, including the loss of electrical conductivity and the decrease in pore access over time.

**Figure 8:**
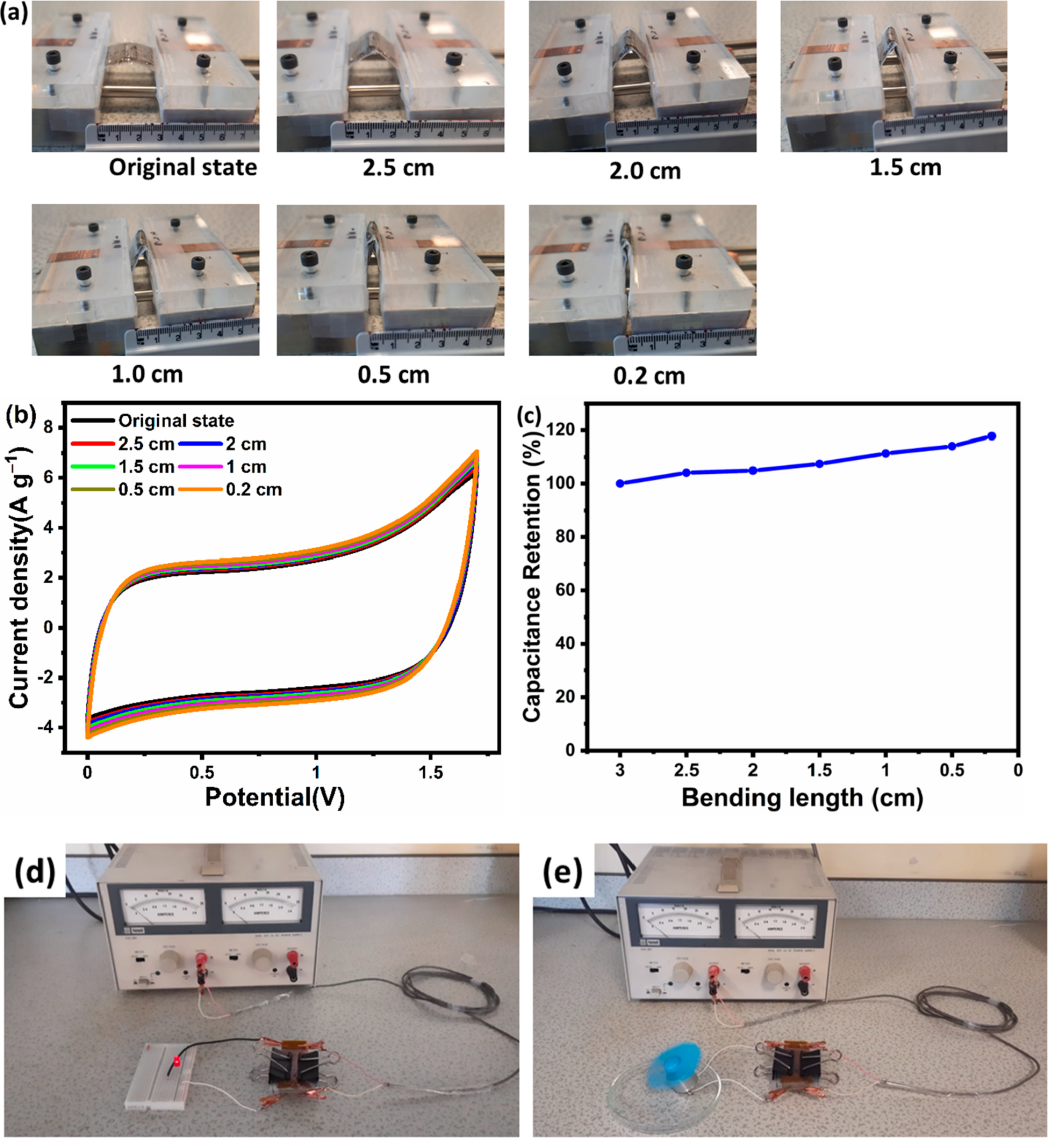
(a) Photographs of the flexible supercapacitor at different bending distances. (b) CV curves of the flexible supercapacitor at different bending distances measured at a scan rate of 25 mV s^–1^. (c) Capacitance retention of the supercapacitor at different bending positions. Photographs of a (d) red LED and (e) motor being powered by the flexible supercapacitor.

In addition to having excellent electrochemical performance during normal operation, it is important for flexible supercapacitor devices to be able to withstand deformation, such as bending. Thus, the mechanical reliability of the device was tested by attaching the two ends of the device to a clamping system (with one end fixed and the other end movable), as shown in Figure 8a. The CV readings were taken after moving one end of the clamp by 0.5 cm toward the fixed end. As indicated in Figure 8b, the CV curves were able to maintain their rectangular shape at different bending distances (separation between the clamps) without any obvious deviation in behavior, indicating that bending the device had no significant impact on its electrochemical performance. As shown in Figure 8c, there was no decay in the capacitance of the device. Indeed, the capacitance actually increased with an increase in the amount of bending due to a lower interfacial resistance. These results indicate that the flexible supercapacitor that was fabricated here has excellent mechanical and electrochemical stability, which suggests the huge potential for the synthesized activated carbon material for use in flexible and portable energy storage devices. The practical realization of this AC grain electrode-based flexible supercapacitor was demonstrated with the help of a light-emitting diode (LED) and a motor. With a charging time of 1 min, the 1.7 V flexible supercapacitor was able to successfully power a red LED and a motor (Figure 8d,e) for nearly 5 min, which signifies the practical application of this flexible supercapacitor in energy storage.

## 4 Conclusions

This work has reported the synthesis of activated carbons from four types of corn derivatives via carbonization and KHCO_3_ activation and, subsequently, their application as supercapacitor electrodes. Out of the four samples, the activated carbon derived from corn grains (AC grain) exhibited the best electrochemical performance in a 1 M Na_2_SO_4_ electrolyte due to its high SSA (1804 m^2^ g^–1^), high degree of graphitization, and appropriate amount of O and N atoms (this material exhibited a high specific capacitance of 385 F g^–1^ at 0.25 A g^–1^ and a capacitance retention of 70.03% at 30 A g^–1^). Moreover, the AC grain electrode was tested in two different electrolytes, and it displayed a high specific capacitance (411 F g^–1^ at 1.0 A g^–1^) and an excellent rate capability (85.7% capacitance retention at 30 A g^–1^) in the 6 M KOH aqueous electrolyte when tested in a three-electrode configuration. In the two-electrode device testing, the assembled flexible supercapacitor device that employed AC grain electrodes exhibited a superior energy density (31.1 Wh kg^–1^) and power density (215 W kg^–1^) over an extended potential window of 1.7 V, and its power density reached 28.01 kW kg^–1^ when the energy density was 20.03 Wh kg^–1^. This flexible supercapacitor also had an excellent cycling stability, as it achieved a small capacitance loss of only 8.7% compared to the first cycle after 10 000 cycles. According to the findings of this study, it is possible to create porous activated carbon from inexpensive corn grains, which is a promising method for creating environmentally friendly, renewable electrode materials for use in high-performance, flexible supercapacitors.

## Supporting Information Available

Deconvoluted Raman spectra; FESEM images; HRTEM images; deconvoluted C 1s, N 1s, and O 1s XPS core-level spectra; contact angle measurements of the AC husk, AC cob, AC fiber, and AC grain samples; equivalent circuit models used for fitting the EIS data; and CV curves for the flexible supercapacitor prepared with the AC grain sample and HEC/KOH electrolyte (PDF)

## References

[ref1] Fagiolari L.; Sampò M.; Lamberti A.; Amici J.; Francia C.; Bodoardo S.; Bella F. Integrated energy conversion and storage devices: interfacing solar cells, batteries and supercapacitors. Energy Storage Materials 2022, 51, 400–434. 10.1016/j.ensm.2022.06.051.

[ref2] Wang Y.; Wu X.; Han Y.; Li T. Flexible supercapacitor: overview and outlooks. Journal of Energy Storage 2021, 42, 103053 10.1016/j.est.2021.103053.

[ref3] Xie P.; Yuan W.; Liu X.; Peng Y.; Yin Y.; Li Y.; Wu Z. Advanced carbon nanomaterials for state-of-the-art flexible supercapacitors. Energy Storage Materials 2021, 36, 56–76. 10.1016/j.ensm.2020.12.011.

[ref4] Delbari S. A.; Ghadimi L. S.; Hadi R.; Farhoudian S.; Nedaei M.; Babapoor A.; Sabahi Namini A.; Le Q. V.; Shokouhimehr M.; Shahedi Asl M.; Mohammadi M. Transition metal oxide-based electrode materials for flexible supercapacitors: A review. J. Alloys Compd. 2021, 857, 158281 10.1016/j.jallcom.2020.158281.

[ref5] Wang Y.; Zhang L.; Hou H.; Xu W.; Duan G.; He S.; Liu K.; Jiang S. Recent progress in carbon-based materials for supercapacitor electrodes: a review. J. Mater. Sci. 2021, 56 (1), 173–200. 10.1007/s10853-020-05157-6.

[ref6] Saini S.; Chand P.; Joshi A. Biomass derived carbon for supercapacitor applications. Journal of Energy Storage 2021, 39, 102646 10.1016/j.est.2021.102646.

[ref7] Zhang H.; Zhang Y.; Bai L.; Zhang Y.; Sun L. Effect of physiochemical properties in biomass-derived materials caused by different synthesis methods and their electrochemical properties in supercapacitors. Journal of Materials Chemistry A 2021, 9 (21), 12521–12552. 10.1039/D1TA00790D.

[ref8] Bai J.; Mao S.; Guo F.; Shu R.; Liu S.; Dong K.; Yu Y.; Qian L. Rapeseed meal-derived N, S self-codoped porous carbon materials for supercapacitors. New J. Chem. 2022, 46 (22), 10752–10764. 10.1039/D2NJ00791F.

[ref9] Lin X.; Xu Y.; Wu J.; Huang J. Bio-inspired hierarchical nanoporous carbon derived from water spinach for high-performance supercapacitor electrode materials. Nanoscale Advances 2022, 4 (5), 1445–1454. 10.1039/D1NA00636C. 36133677 PMC9417308

[ref10] Cheng J.; Hu S.-C.; Sun G.-T.; Kang K.; Zhu M.-Q.; Geng Z.-C. Comparison of activated carbons prepared by one-step and two-step chemical activation process based on cotton stalk for supercapacitors application. Energy 2021, 215, 119144 10.1016/j.energy.2020.119144.

[ref11] Bhattarai R. M.; Chhetri K.; Natarajan S.; Saud S.; Kim S. J.; Mok Y. S. Activated carbon derived from cherry flower biowaste with a self-doped heteroatom and large specific surface area for supercapacitor and sodium-ion battery applications. Chemosphere 2022, 303, 135290 10.1016/j.chemosphere.2022.135290. 35691391

[ref12] Lu S.; Yang W.; Zhou M.; Qiu L.; Tao B.; Zhao Q.; Wang X.; Zhang L.; Xie Q.; Ruan Y. Nitrogen-and oxygen-doped carbon with abundant micropores derived from biomass waste for all-solid-state flexible supercapacitors. J. Colloid Interface Sci. 2022, 610, 1088–1099. 10.1016/j.jcis.2021.11.164. 34876262

[ref13] Mo R.-J.; Zhao Y.; Wu M.; Xiao H.-M.; Kuga S.; Huang Y.; Li J.-P.; Fu S.-Y. Activated carbon from nitrogen rich watermelon rind for high-performance supercapacitors. RSC Adv. 2016, 6 (64), 59333–59342. 10.1039/C6RA10719B.

[ref14] Momodu D.; Bello A.; Oyedotun K.; Ochai-Ejeh F.; Dangbegnon J.; Madito M.; Manyala N. Enhanced electrochemical response of activated carbon nanostructures from tree-bark biomass waste in polymer-gel active electrolytes. Rsc Advances 2017, 7 (59), 37286–37295. 10.1039/C7RA05810A.

[ref15] Miranda M.; Sepúlveda F.; Arranz J.; Montero I.; Rojas C. Analysis of pelletizing from corn cob waste. Journal of environmental management 2018, 228, 303–311. 10.1016/j.jenvman.2018.08.105. 30236883

[ref16] Ponce J.; Andrade J. G. d. S.; dos Santos L. N.; Bulla M. K.; Barros B. C. B.; Favaro S. L.; Hioka N.; Caetano W.; Batistela V. R. Alkali pretreated sugarcane bagasse, rice husk and corn husk wastes as lignocellulosic biosorbents for dyes. Carbohydrate Polymer Technologies and Applications 2021, 2, 100061 10.1016/j.carpta.2021.100061.

[ref17] Usha Rani M.; Nanaji K.; Rao T. N.; Deshpande A. S. Corn husk derived activated carbon with enhanced electrochemical performance for high-voltage supercapacitors. J. Power Sources 2020, 471, 228387 10.1016/j.jpowsour.2020.228387.

[ref18] Yang S.; Zhang K. Converting corncob to activated porous carbon for supercapacitor application. Nanomaterials 2018, 8 (4), 181 10.3390/nano8040181. 29561807 PMC5923511

[ref19] Karnan M.; Subramani K.; Srividhya P.; Sathish M. Electrochemical studies on corncob derived activated porous carbon for supercapacitors application in aqueous and non-aqueous electrolytes. Electrochim. Acta 2017, 228, 586–596. 10.1016/j.electacta.2017.01.095.

[ref20] Wang F.; Zheng F.; Jiang J.; Li Y.; Luo Y.; Chen K.; Du J.; Huang Y.; Li Q.; Wang H. Microwave-assisted preparation of hierarchical N and O co-doped corn-cob-derived activated carbon for a high-performance supercapacitor. Energy Fuels 2021, 35 (9), 8334–8344. 10.1021/acs.energyfuels.1c00337.

[ref21] Yue X.; Yang H.; Cao Y.; Jiang L.; Li H.; Shi F.; Liu J. Nitrogen-doped cornstalk-based biomass porous carbon with uniform hierarchical pores for high-performance symmetric supercapacitors. J. Mater. Sci. 2022, 57 (5), 3645–3661. 10.1007/s10853-022-06891-9.

[ref22] Balathanigaimani M.; Shim W.-G.; Lee M.-J.; Kim C.; Lee J.-W.; Moon H. Highly porous electrodes from novel corn grains-based activated carbons for electrical double layer capacitors. Electrochem. Commun. 2008, 10 (6), 868–871. 10.1016/j.elecom.2008.04.003.

[ref23] Shell K. M.; Rodene D. D.; Amar V.; Thakkar A.; Maddipudi B.; Kumar S.; Shende R.; Gupta R. B. Supercapacitor performance of corn stover-derived biocarbon produced from the solid co-products of a hydrothermal liquefaction process. Bioresource Technology Reports 2021, 13, 100625 10.1016/j.biteb.2021.100625.

[ref24] Yu K.; Zhu H.; Qi H.; Liang C. High surface area carbon materials derived from corn stalk core as electrode for supercapacitor. Diamond Relat. Mater. 2018, 88, 18–22. 10.1016/j.diamond.2018.06.018.

[ref25] Shi G.; Zhang H.; Dong Y.; Zhang Q.; Wang Z.; Jiang X.; Hu Y.; Luo F.; Li X.; Wang G. Preparation and Activation of Corn Straw-Based Carbon and Its Application in Supercapacitors. INTERNATIONAL JOURNAL OF ELECTROCHEMICAL SCIENCE 2019, 14 (8), 7608–7622. 10.20964/2019.08.38.

[ref26] Yu H.; Zhang W.; Li T.; Zhi L.; Dang L.; Liu Z.; Lei Z. Capacitive performance of porous carbon nanosheets derived from biomass cornstalk. RSC Adv. 2017, 7 (2), 1067–1074. 10.1039/C6RA25899A.

[ref27] Zhong C.; Deng Y.; Hu W.; Qiao J.; Zhang L.; Zhang J. A review of electrolyte materials and compositions for electrochemical supercapacitors. Chem. Soc. Rev. 2015, 44 (21), 7484–7539. 10.1039/C5CS00303B. 26050756

[ref28] Zheng Y.; Wang D.; Kaushik S.; Zhang S.; Wada T.; Hwang J.; Matsumoto K.; Hagiwara R. Ionic liquid electrolytes for next-generation electrochemical energy devices. EnergyChem. 2022, 4 (3), 100075 10.1016/j.enchem.2022.100075.

[ref29] Xu C.; Yang G.; Wu D.; Yao M.; Xing C.; Zhang J.; Zhang H.; Li F.; Feng Y.; Qi S.; Zhuo M.; Ma J. Roadmap on ionic liquid electrolytes for energy storage devices. Chemistry An Asian Journal 2021, 16 (6), 549–562. 10.1002/asia.202001414. 33377601

[ref30] Pan S.; Yao M.; Zhang J.; Li B.; Xing C.; Song X.; Su P.; Zhang H. Recognition of ionic liquids as high-voltage electrolytes for supercapacitors. Frontiers in Chemistry 2020, 8, 261 10.3389/fchem.2020.00261. 32432074 PMC7214745

[ref31] Xu T.; Liu K.; Sheng N.; Zhang M.; Liu W.; Liu H.; Dai L.; Zhang X.; Si C.; Du H.; Zhang K. Biopolymer-based hydrogel electrolytes for advanced energy storage/conversion devices: Properties, applications, and perspectives. Energy Storage Materials 2022, 48, 244–262. 10.1016/j.ensm.2022.03.013.

[ref32] Adhikari M. P.; Adhikari R.; Shrestha R. G.; Rajendran R.; Adhikari L.; Bairi P.; Pradhananga R. R.; Shrestha L. K.; Ariga K. Nanoporous activated carbons derived from agro-waste corncob for enhanced electrochemical and sensing performance. Bull. Chem. Soc. Jpn. 2015, 88 (8), 1108–1115. 10.1246/bcsj.20150092.

[ref33] Pang L.; Zou B.; Zou Y.; Han X.; Cao L.; Wang W.; Guo Y. A new route for the fabrication of corn starch-based porous carbon as electrochemical supercapacitor electrode material. Colloids Surf., A 2016, 504, 26–33. 10.1016/j.colsurfa.2016.05.049.

[ref34] Reddygunta K. K. R.; Callander A.; Šiller L.; Faulds K.; Berlouis L.; Ivaturi A. Sono-exfoliated graphene-like activated carbon from hazelnut shells for flexible supercapacitors. International Journal of Energy Research 2022, 46 (12), 16512–16537. 10.1002/er.8314.

[ref35] Mutuma B. K.; Matsoso B. J.; Momodu D.; Oyedotun K. O.; Coville N. J.; Manyala N. Deciphering the structural, textural, and electrochemical properties of activated BN-doped spherical carbons. Nanomaterials 2019, 9 (3), 446 10.3390/nano9030446. 30884783 PMC6474088

[ref36] Sivachidambaram M.; Vijaya J. J.; Kennedy L. J.; Jothiramalingam R.; Al-Lohedan H. A.; Munusamy M. A.; Elanthamilan E.; Merlin J. P. Preparation and characterization of activated carbon derived from the Borassus flabellifer flower as an electrode material for supercapacitor applications. New J. Chem. 2017, 41 (10), 3939–3949. 10.1039/C6NJ03867K.

[ref37] Sun Z.; Zheng M.; Hu H.; Dong H.; Liang Y.; Xiao Y.; Lei B.; Liu Y. From biomass wastes to vertically aligned graphene nanosheet arrays: a catalyst-free synthetic strategy towards high-quality graphene for electrochemical energy storage. Chemical Engineering Journal 2018, 336, 550–561. 10.1016/j.cej.2017.12.019.

[ref38] Gupta G. K.; Sagar P.; Pandey S. K.; Srivastava M.; Singh A. K.; Singh J.; Srivastava A.; Srivastava S. K.; Srivastava A. In Situ fabrication of activated carbon from a bio-waste Desmostachya bipinnata for the improved supercapacitor performance. Nanoscale Res. Lett. 2021, 16, 85 10.1186/s11671-021-03545-8. 33987738 PMC8119520

[ref39] Wang Y.; Yang B.; Zhang D.; Shi H.; Lei M.; Li H.; Wang K. Strong polar nonaqueous solvent-assisted microwave fabrication of N and P co-doped microporous carbon for high-performance supercapacitor. Appl. Surf. Sci. 2020, 512, 145711 10.1016/j.apsusc.2020.145711.

[ref40] Zhang D.; Yang B.; She W.; Gao S.; Wang J.; Wang Y.; Wang K.; Li H.; Han L. Simultaneously achieving high energy and power density for ultrafast-charging supercapacitor built by a semi-graphitic hierarchical porous carbon nanosheet and a high-voltage alkaline aqueous electrolyte. J. Power Sources 2021, 506, 230103 10.1016/j.jpowsour.2021.230103.

[ref41] Shi L.; Jin L.; Meng Z.; Sun Y.; Li C.; Shen Y. A novel porous carbon material derived from the byproducts of bean curd stick manufacture for high-performance supercapacitor use. RSC Adv. 2018, 8 (70), 39937–39947. 10.1039/C8RA08664H. 35558204 PMC9091489

[ref42] Jain D.; Kanungo J.; Tripathi S. Enhancement in performance of supercapacitor using eucalyptus leaves derived activated carbon electrode with CH3COONa and HQ electrolytes: A step towards environment benign supercapacitor. J. Alloys Compd. 2020, 832, 154956 10.1016/j.jallcom.2020.154956.

[ref43] Ran F.; Yang X.; Xu X.; Li S.; Liu Y.; Shao L. Green activation of sustainable resources to synthesize nitrogen-doped oxygen-riched porous carbon nanosheets towards high-performance supercapacitor. Chemical Engineering Journal 2021, 412, 128673 10.1016/j.cej.2021.128673.

[ref44] Hamouda H. A.; Cui S.; Dai X.; Xiao L.; Xie X.; Peng H.; Ma G. Synthesis of porous carbon material based on biomass derived from hibiscus sabdariffa fruits as active electrodes for high-performance symmetric supercapacitors. RSC Adv. 2021, 11 (1), 354–363. 10.1039/D0RA09509E. PMC8691107 35423056

[ref45] Karnan M.; Subramani K.; Sudhan N.; Ilayaraja N.; Sathish M. Aloe vera derived activated high-surface-area carbon for flexible and high-energy supercapacitors. ACS Appl. Mater. Interfaces 2016, 8 (51), 35191–35202. 10.1021/acsami.6b10704. 27977134

[ref46] Lu X.; Xiang K.; Zhou W.; Zhu Y.; Chen H. Biomass carbon materials derived from macadamia nut shells for high-performance supercapacitors. Bulletin of Materials Science 2018, 41, 138 10.1007/s12034-018-1666-3.

[ref47] Nath G.; Singh P. K.; Dhapola P. S.; Dohare S.; Noor I. M.; Sharma T.; Singh A. Fabrication of cornstarch biopolymer-derived nano porous carbon as electrode material for supercapacitor application. Biomass Conversion and Biorefinery 2022, 10.1007/s13399-022-02656-1.

[ref48] Wu X.; Tian Z.; Hu L.; Huang S.; Cai J. Macroalgae-derived nitrogen-doped hierarchical porous carbons with high performance for H 2 storage and supercapacitors. RSC Adv. 2017, 7 (52), 32795–32805. 10.1039/C7RA05355J.

[ref49] Qiang L.; Hu Z.; Li Z.; Yang Y.; Wang X.; Zhou Y.; Zhang X.; Wang W.; Wang Q. Hierarchical porous biomass carbon derived from cypress coats for high energy supercapacitors. Journal of Materials Science: Materials in Electronics 2019, 30 (8), 7324–7336. 10.1007/s10854-019-01045-1.

[ref50] Chen X.; Zhang J.; Zhang B.; Dong S.; Guo X.; Mu X.; Fei B. A novel hierarchical porous nitrogen-doped carbon derived from bamboo shoot for high performance supercapacitor. Sci. Rep. 2017, 7, 7362 10.1038/s41598-017-06730-x. 28779072 PMC5544758

[ref51] Yan S.; Lin J.; Liu P.; Zhao Z.; Lian J.; Chang W.; Yao L.; Liu Y.; Lin H.; Han S. Preparation of nitrogen-doped porous carbons for high-performance supercapacitor using biomass of waste lotus stems. RSC Adv. 2018, 8 (13), 6806–6813. 10.1039/C7RA13013A. 35540345 PMC9078325

[ref52] Yuan Y.; Sun Y.; Feng Z.; Li X.; Yi R.; Sun W.; Zhao C.; Yang L. Nitrogen-doped hierarchical porous activated carbon derived from paddy for high-performance supercapacitors. Materials 2021, 14 (2), 318 10.3390/ma14020318. 33435436 PMC7828036

[ref53] Chen H.; Yu F.; Wang G.; Chen L.; Dai B.; Peng S. Nitrogen and sulfur self-doped activated carbon directly derived from elm flower for high-performance supercapacitors. ACS omega 2018, 3 (4), 4724–4732. 10.1021/acsomega.8b00210. 30023900 PMC6045337

[ref54] Shard A. G. Practical guides for x-ray photoelectron spectroscopy: Quantitative XPS. J. Vac. Sci. Technol. A 2020, 38, 041201 10.1116/1.5141395.

[ref55] Feng H.; Hu H.; Dong H.; Xiao Y.; Cai Y.; Lei B.; Liu Y.; Zheng M. Hierarchical structured carbon derived from bagasse wastes: A simple and efficient synthesis route and its improved electrochemical properties for high-performance supercapacitors. J. Power Sources 2016, 302, 164–173. 10.1016/j.jpowsour.2015.10.063.

[ref56] Fic K.; Lota G.; Meller M.; Frackowiak E. Novel insight into neutral medium as electrolyte for high-voltage supercapacitors. Energy Environ. Sci. 2012, 5 (2), 5842–5850. 10.1039/C1EE02262H.

[ref57] Gandla D.; Wu X.; Zhang F.; Wu C.; Tan D. Q. High-performance and high-voltage supercapacitors based on N-doped mesoporous activated carbon derived from dragon fruit peels. ACS omega 2021, 6 (11), 7615–7625. 10.1021/acsomega.0c06171. 33778272 PMC7992145

[ref58] Chen Z.; Wang X.; Ding Z.; Wei Q.; Wang Z.; Yang X.; Qiu J. Biomass-based Hierarchical Porous Carbon for Supercapacitors: Effect of Aqueous and Organic Electrolytes on the Electrochemical Performance. ChemSusChem 2019, 12 (23), 5099–5110. 10.1002/cssc.201902218. 31612622

[ref59] Liu Y.; Tan H.; Tan Z.; Cheng X. Rice husk derived capacitive carbon prepared by one-step molten salt carbonization for supercapacitors. Journal of Energy Storage 2022, 55, 105437 10.1016/j.est.2022.105437.

