# Supplementary material for: Activated Carbon Utilization from Corn Derivatives for High-Energy-Density Flexible Supercapacitors
Source: Energy Fuels. 2023 Nov 22;37(23):19248–65. doi: 10.1021/acs.energyfuels.3c01925 (PMC10714350; doi:10.1021/acs.energyfuels.3c01925)
Supplement: Supplementary file 1 — ef3c01925_si_001.pdf [file ef3c01925_si_001.pdf]

Supporting Information

Activated Carbon Utilization from Corn Derivatives for High Energy Density Flexible Supercapacitors

*Kiran Kumar Reddy Reddygunta,[a] Rachael Beresford,[a] Lidija Šiller,[b] Leonard Berlouis,[a] and Aruna Ivaturi[a]**

[a]Smart Materials Research and Device Technology (SMaRDT) Group, Department of Pure and Applied Chemistry, University of Strathclyde, Thomas Graham Building, Glasgow, G1 1XL, UK

[b]School of Engineering, Newcastle University, Newcastle upon Tyne, NE1 7RU, UK

Corresponding author email: aruna.ivaturi@strath.ac.uk

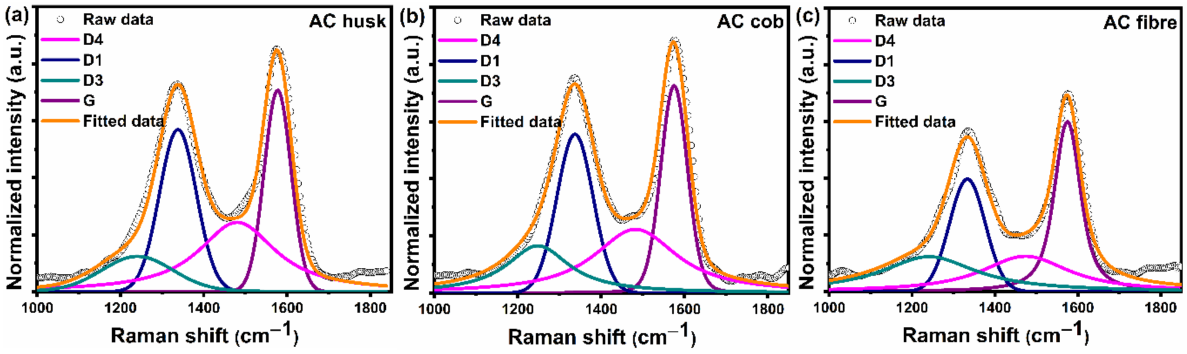

Figure S1. Deconvoluted Raman spectra of (a) AC husk (b) AC cob (c) AC fibre samples performed using Fityk software

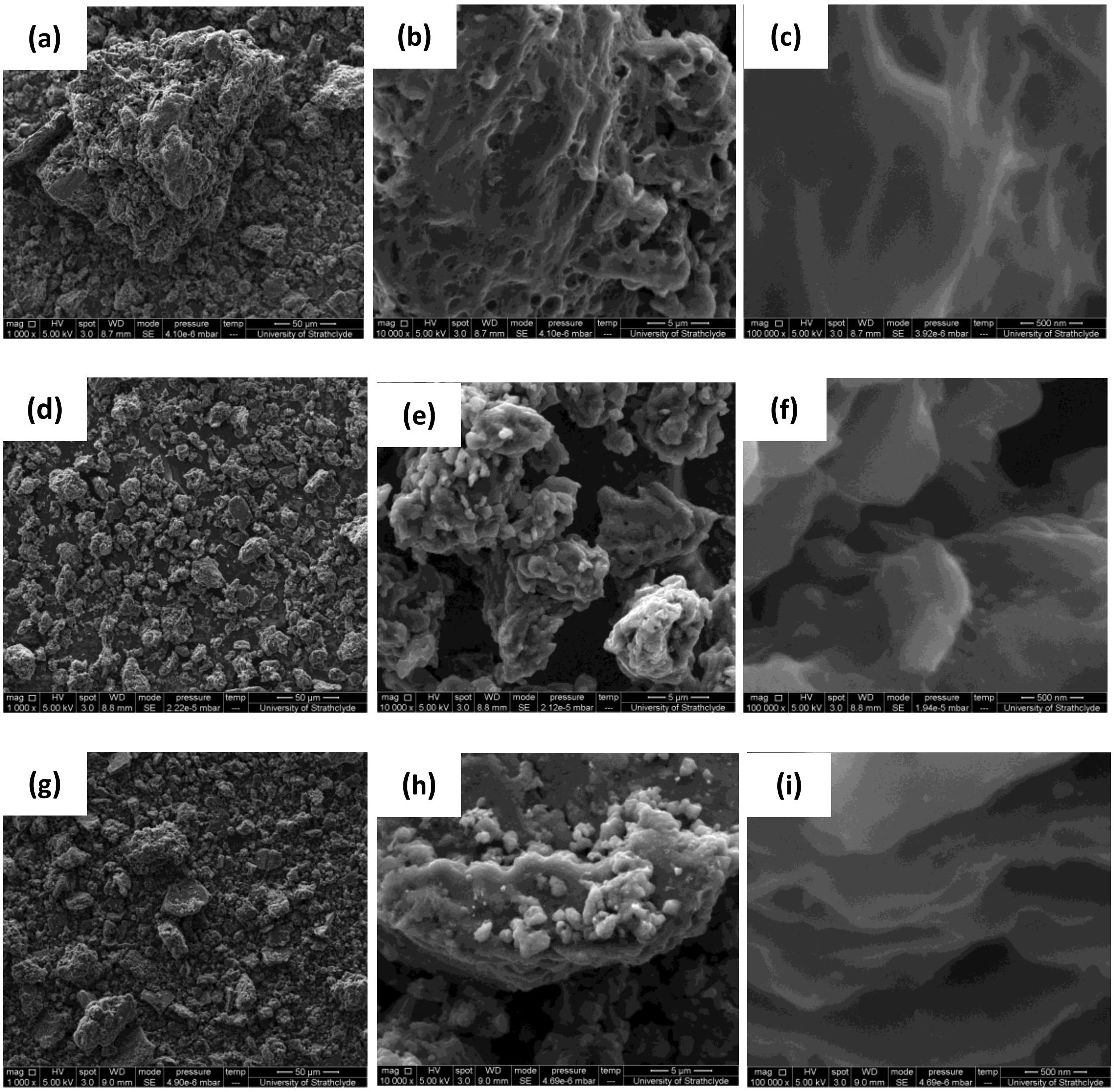

Figure S1. FESEM images of corn based activated carbons (a-c) AC husk (d-f) AC cob (g-i) AC fibre

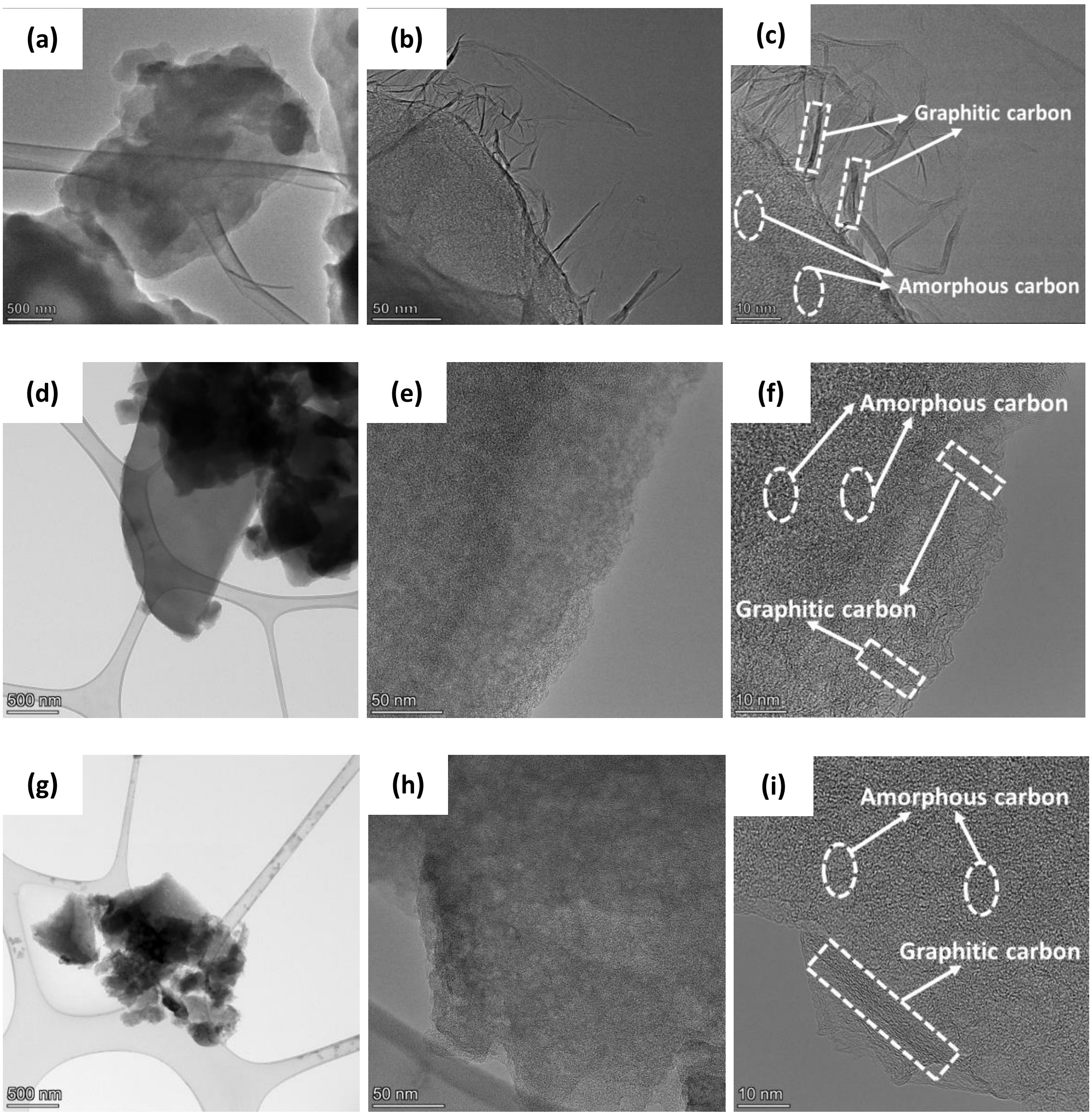

Figure S3. HRTEM images of corn based activated carbons (a-c) AC husk (d-f) AC cob (g-i) AC fibre

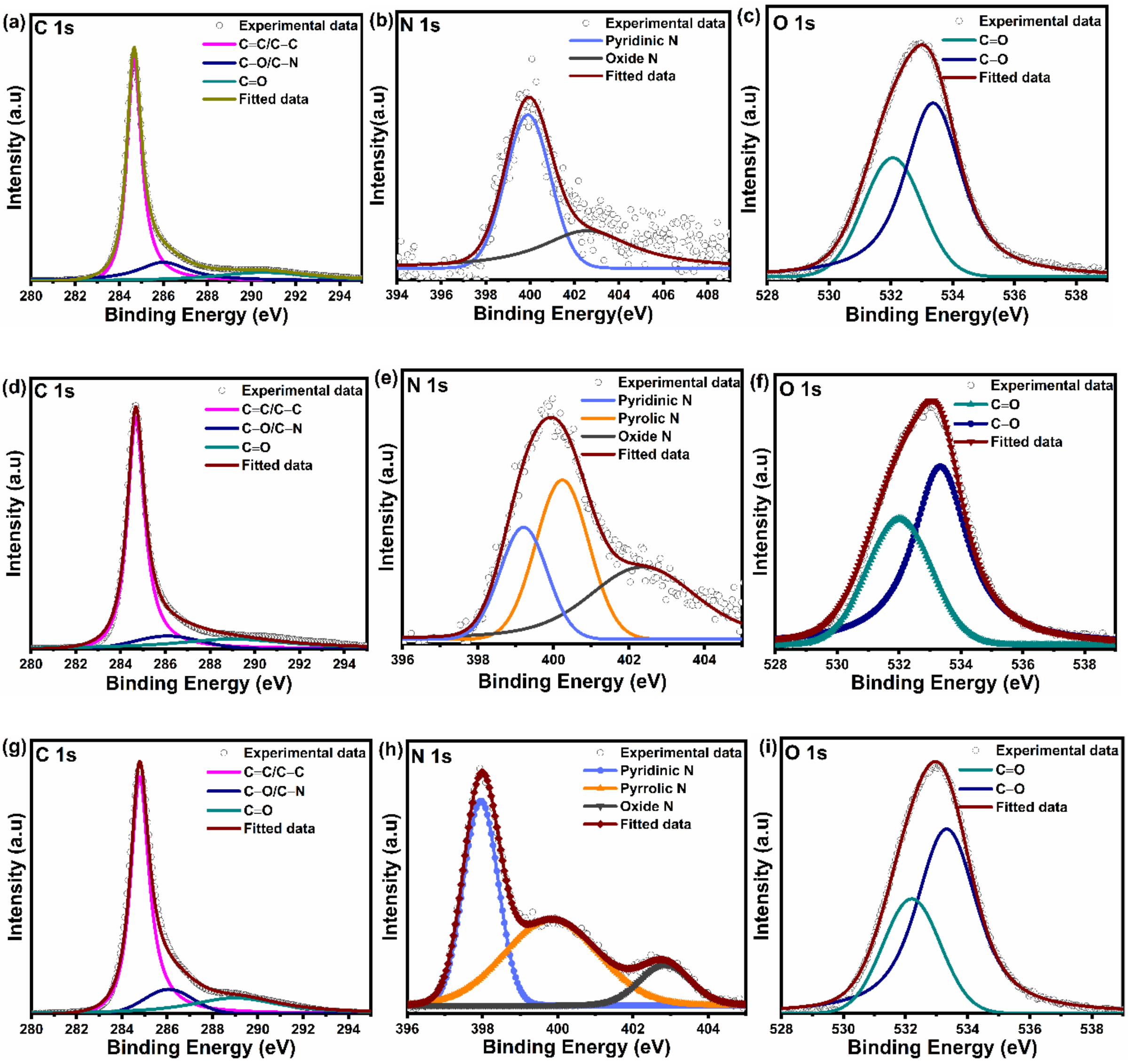

Figure S4. Deconvoluted C1s, N1s and O1s XPS core level spectra of (a-c) AC husk sample (d-f) AC cob sample and (g-i) AC fibre sample

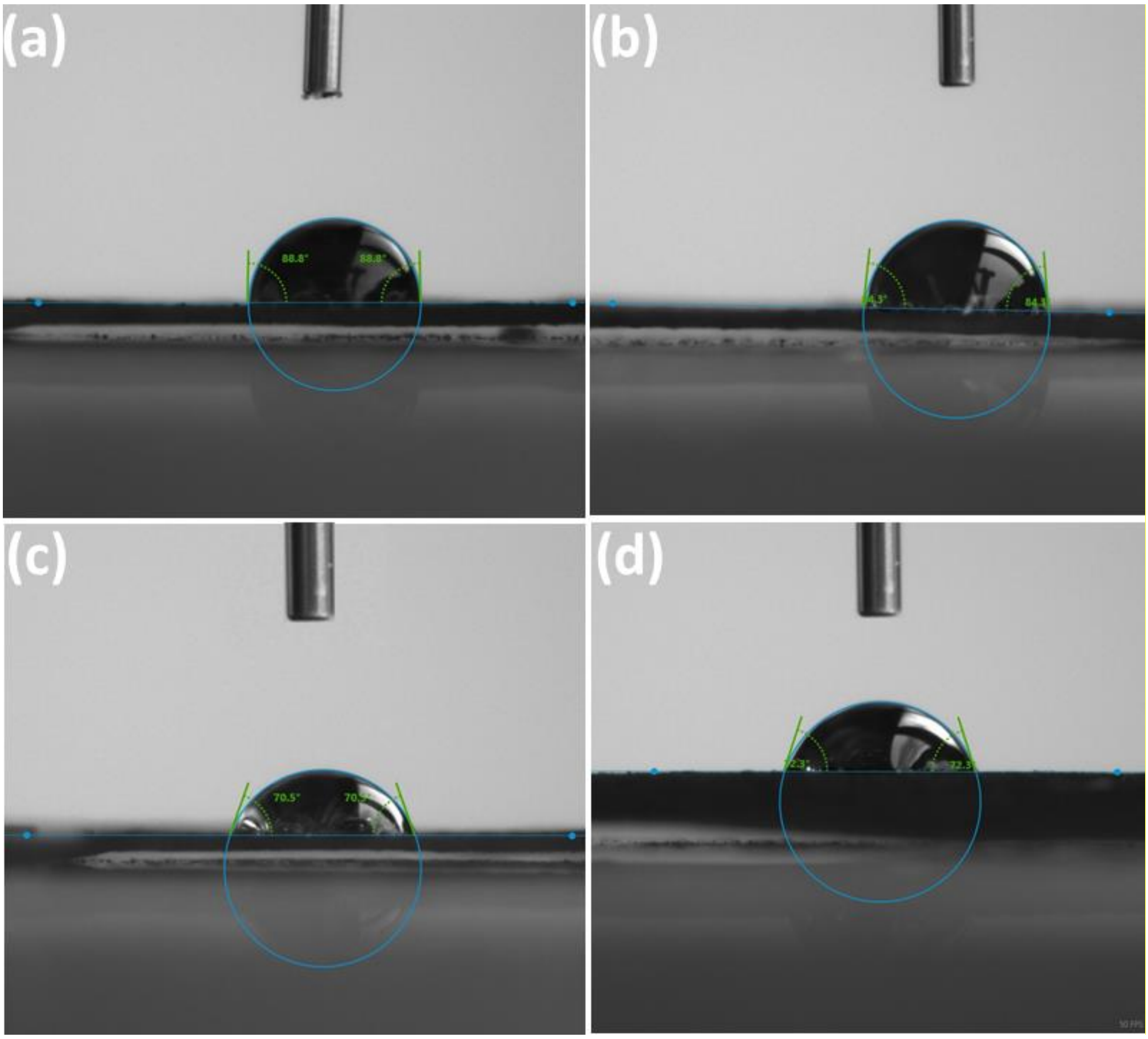

Figure S5. Contact angle measurements of different corn based activated carbons (a) AC husk (b) AC cob (c) AC fibre (d) AC grain electrodes

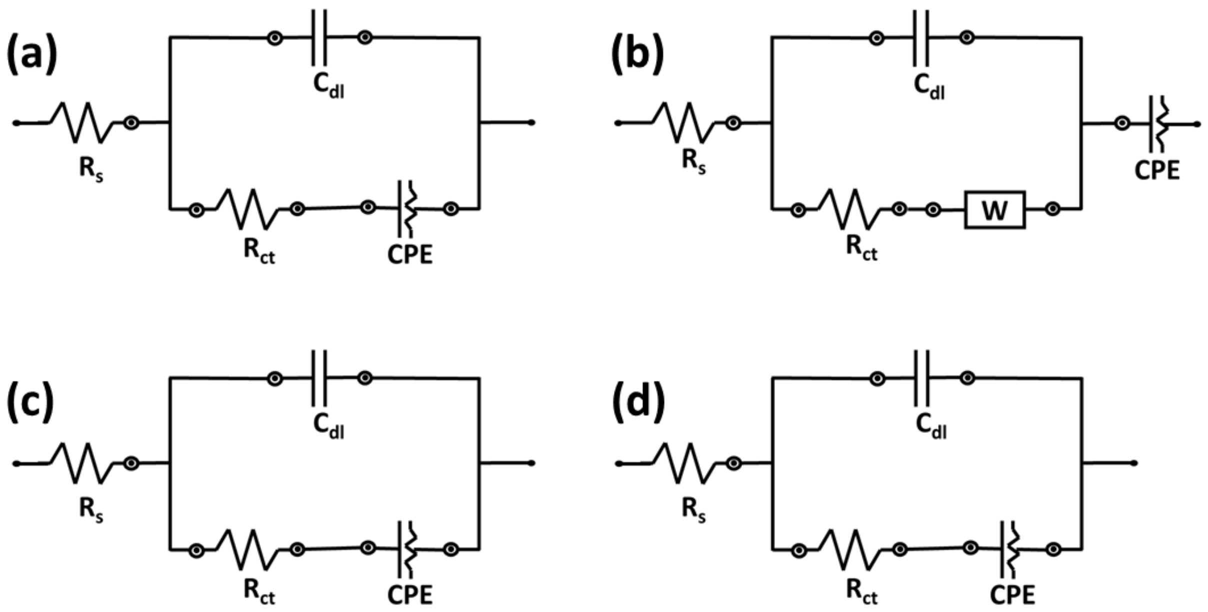

Figure S6. (a) Equivalent circuit model of AC grain (b) Equivalent circuit model of AC fibre (c) Equivalent circuit model of AC cob (d) Equivalent circuit model of AC husk samples in 1 M Na_2SO_4 electrolyte

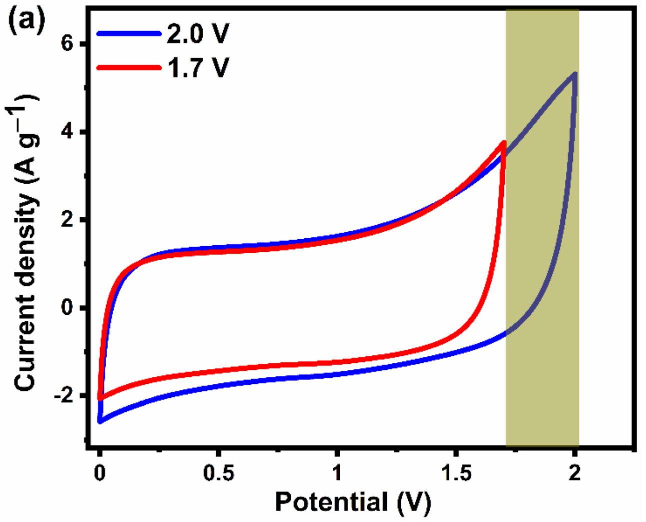

Figure S7. (a) CV curve measured at 10 mV s^{-1} over 1.7 and 2.0 V voltage range for a flexible supercapacitor prepared with AC grain sample and HEC/KOH electrolyte
